# Low-loss metasurface optics down to the deep ultraviolet region

**DOI:** 10.1038/s41377-020-0287-y

**Published:** 2020-04-09

**Authors:** Cheng Zhang, Shawn Divitt, Qingbin Fan, Wenqi Zhu, Amit Agrawal, Yanqing Lu, Ting Xu, Henri J. Lezec

**Affiliations:** 1 School of Optical and Electronic Information & Wuhan National Laboratory for Optoelectronics, Huazhong University of Science and Technology, 430074 Wuhan, China; 2 Physical Measurement Laboratory, National Institute of Standards and Technology, 20899 Gaithersburg, MD USA; 3 Maryland Nanocenter, University of Maryland, College Park, MD 20742 USA; 4 National Laboratory of Solid State Microstructures, College of Engineering and Applied Sciences and Collaborative Innovation Center of Advanced Microstructures, Nanjing University, 210093 Nanjing, China

**Keywords:** Metamaterials, Integrated optics

## Abstract

Shrinking conventional optical systems to chip-scale dimensions will benefit custom applications in imaging, displaying, sensing, spectroscopy, and metrology. Towards this goal, metasurfaces—planar arrays of subwavelength electromagnetic structures that collectively mimic the functionality of thicker conventional optical elements—have been exploited at frequencies ranging from the microwave range up to the visible range. Here, we demonstrate high-performance metasurface optical components that operate at ultraviolet wavelengths, including wavelengths down to the record-short deep ultraviolet range, and perform representative wavefront shaping functions, namely, high-numerical-aperture lensing, accelerating beam generation, and hologram projection. The constituent nanostructured elements of the metasurfaces are formed of hafnium oxide—a loss-less, high-refractive-index dielectric material deposited using low-temperature atomic layer deposition and patterned using high-aspect-ratio Damascene lithography. This study opens the way towards low-form factor, multifunctional ultraviolet nanophotonic platforms based on flat optical components, enabling diverse applications including lithography, imaging, spectroscopy, and quantum information processing.

## Nanopillars that manipulate UV light

An array of hafnium oxide nanopillars on a fused silica substrate efficiently manipulates a broad range of ultraviolet light wavelengths, with potential applications in photolithography, imaging, spectroscopy, and quantum information processing. A resist-based Damascene process was developed by a team led by Cheng Zhang, Ting Xu and Henri J. Lezec in China and the USA. They used lithography to design spaces into a resist template that were then filled with hafnium oxide. The resist was removed with solvent to leave high-aspect-ratio hafnium oxide nanopillars. The pillar height, diameter and orientation, as well as the spacing between the pillars, control how UV light propagates through the thin ‘metasurface’. Metasurfaces were used to focus UV light like a lens; transform incident UV light into a propagating, curving output beam; and generate holograms from three UV light wavelengths.

## Introduction

An optical metasurface is a planar array of subwavelength electromagnetic structures that emulate the operation of a conventional refractive, birefringent, or diffractive optical component, such as a lens, waveplate, or hologram, through individually tailored amplitude, phase, or polarization transformations of the incident light^1–9^. Dielectric materials such as amorphous Si^10,11^, polycrystalline Si^12^, titanium dioxide (TiO_2_)^13,14^, and gallium nitride (GaN)^15,16^ have been used to realize metasurfaces operating at infrared and visible frequencies. The scarcity of dielectric materials that are characterized by low optical loss at higher frequencies and simultaneously amenable to high-aspect-ratio nanopatterning has impeded the development of metasurfaces for applications in the ultraviolet (UV) range, a technologically important spectral regime hosting diverse applications in lithography, imaging, spectroscopy, time keeping, and quantum information processing^17–19^. To date, metasurfaces designed for operation in the near-UV range (UV-A; free-space wavelength range: 315 nm ≤ *λ*_0_ ≤ 380 nm; energy range: 3.26 eV ≤ *E*_0_ ≤ 3.94 eV) have been implemented using niobium pentoxide (Nb_2_O_5_), down to an operation free-space wavelength of *λ*_0_ = 355 nm^20^. Crystalline Si has been used to realize metasurfaces operating down to *λ*_0_ = 290 nm^21^, a wavelength that falls within the mid-UV range (UV-B; 280 nm ≤ *λ*_0_ ≤ 315 nm; 3.94 eV ≤ *E*_0_ ≤ 4.43 eV), but the device efficiencies remain limited by the severe absorption loss associated with illumination frequencies above the bandgap of Si (*E*_g_ ≈ 1.1 eV). In both studies, the demonstrated functionalities are limited to hologram generation and beam deflection, while other important wavefront shaping functionalities that can be empowered by optical metasurfaces, such as high-numerical-aperture focusing and structured beam generation, have not yet been achieved. Meanwhile, metasurfaces that can operate at even higher frequencies, such as within the deep-UV range (longer wavelength portion of UV-C; 190 nm ≤ *λ*_0_ ≤ 280 nm; 4.43 eV ≤ *E*_0_ ≤ 6.53 eV), have not been realized due to the challenge of identifying a dielectric material that has a suitably low optical absorption coefficient in that range and can be patterned into high-aspect-ratio nanostructures using the available nanofabrication techniques.

Here, we report high-performance dielectric metasurfaces that operate over a broad UV range, including within the record-short, deep-UV regime, and perform representative wavefront shaping functionalities. The constituent nanostructured elements of the metasurfaces are formed of hafnium oxide (HfO_2_)—a UV-transparent, high-refractive-index dielectric material. Although HfO_2_ has been commonly exploited as a high-static-dielectric-constant (high-*κ*) material in integrated circuit fabrication^22,23^, its applications in photonics have largely been limited to optical coatings based on planar thin films, in particular due to the difficulty of patterning the material into high-aspect-ratio nanostructures. In this work, we overcome this limitation and use HfO_2_, for the first time, to implement meta-devices operating in the UV and deep-UV regimes. We deposit high-quality, UV-transparent HfO_2_ films using low-temperature atomic layer deposition (ALD) and pattern the films using a high-aspect ratio, resist-based Damascene lithography technique^24–26^. We implement metasurfaces designed for operation at three representative UV wavelengths of 364, 325, and 266 nm, which perform a variety of optical functions, namely, high-numerical-aperture lensing, accelerating beam generation, and hologram projection, including under spin control for the last two applications. This achievement opens the way for low-form-factor and multifunctional photonic systems based on UV flat optics, and suggests promising applications in photolithography, high-resolution imaging, UV spectroscopy and quantum information processing.

## Results

### Material choice and fabrication approach

**Fig. 1:**
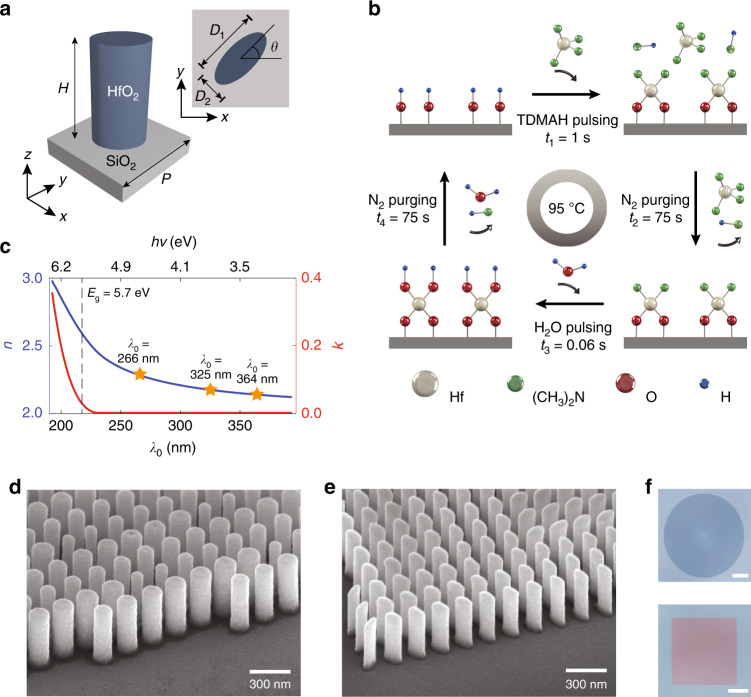
Implementation of ultraviolet metasurfaces. **a** Schematic representation of a metasurface unit cell, consisting of a high-aspect-ratio HfO_2_ pillar with height *H*, an elliptical cross-section (principle axis lengths *D*_1_ and *D*_2_), and rotation angle *θ*, arranged on a SiO_2_ substrate to form a square lattice with a subwavelength lattice spacing *P*. Specific optical functions are implemented via the variation in *D*_1_, *D*_2_, and *θ* as a function of the nanopillar position within the lattice. **b** Schematic representation of the developed low-temperature ALD cycle using the TDMAH precursor, H_2_O reactant, and a process temperature of *T*_p_ = 95 °C. **c** Refractive index *n* and extinction coefficient *k* of the as-deposited HfO_2_ film, measured using spectroscopic ellipsometry. The values of *n* at the three operation wavelengths targeted in this study are denoted by yellow stars. The dashed line indicates the position of the HfO_2_ bandgap *E*_g_. **d** Scanning electron microscopy (SEM) image of the details of a fabricated polarization-independent metalens designed for operation at *λ*_0_ = 325 nm, showing a lattice of 500 nm tall, circularly shaped HfO_2_ nanopillars with various diameters. The viewing angle is 52°. **e** SEM image of the details of a fabricated spin-multiplexed metahologram designed for operation at *λ*_0_ = 266 nm, showing a lattice of 480 nm tall, elliptically shaped HfO_2_ nanopillars with various in-plane cross-sections and rotation angles. The viewing angle is 52°. The nanopillars are coated with a layer of Au/Pd alloy (≈5 nm thick) to suppress charging during SEM imaging. **f** Optical micrographs of the full metalens (top panel) and spin-multiplexed metahologram (bottom panel) corresponding to the metasurfaces described in **d**, **e**, respectively. Scale bars: 100 µm

The implemented metasurface devices consist of HfO_2_ nanopillars of either circular or elliptical in-plane cross-sections (Fig. 1a), densely arrayed on a transparent UV-grade fused silica substrate with a low refractive index (Supplementary Information, Fig. S1). The choice of HfO_2_—a material most commonly exploited for its high static dielectric constant as a transistor gate insulator in complementary metal oxide semiconductor (CMOS) integrated circuits—is guided by the promise of both a large refractive index (*n* > 2.1 for *λ*_0_ < 400 nm) and a wide bandgap *E*_g_ = 5.7 eV (*λ*_g_ = 217 nm) located well within the deep-UV range, leading to a negligible extinction coefficient (*k* ≈ 0) for *λ*_0_ ≥ *λ*_g_. Although the requirement of nanopillar dimensions with a wavelength-scale height (several hundred nanometers), a subwavelength-scale in-plane circle diameter or ellipse minor axis (few tens of nanometers), and vertical sidewalls suggest that pattern transfer with a directional dry-etching technique such as reactive ion etching would be optimal, we were unable to identify a suitable high-aspect-ratio, dry-etching chemistry for HfO_2_ (a material commonly patterned by nondirectional wet chemical etching^27^). We instead explore the use of Damascene lithography^24–26^ for HfO_2_ metasurface fabrication, a process that involves first patterning resist using electron beam (e-beam) lithography, conformally filling the open volumes of the resist template with HfO_2_ using ALD, back-etching the over-coated HfO_2_ layer using argon (Ar) ion milling, and finally removing the remaining resist with solvent to form the required high-aspect-ratio nanopillars (see “Materials and methods”).

The preservation of the physical integrity of the resist template requires the use of a plasma-free thermal ALD process with a process temperature, *T*_p_, lower than the glass transition temperature (reflow temperature) of the utilized resist, *T*_g_, along with a process chemistry involving by-products that are not corrosive to the resist. Fulfilling both process tolerance requirements rules out the use of common Hf precursors such as Tetrakis(ethylmethylamino)Hafnium (TEMAH)^28^, for which the minimum *T*_p_ (≈150 °C) is significantly higher than the *T*_g_ of common e-beam resists, or hafnium chloride (HfCl_4_)^29^, for which the reaction by-product (HCl) attacks the resist. Instead, we investigate Tetrakis(dimethylamino)Hafnium (TDMAH)^30^ as an alternative Hf precursor for thermal ALD of high-optical-quality HfO_2_, using a *T*_p_ below the *T*_g_ of common e-beam organic resists (such as ZEP, for which *T*_g_ ≈ 105 °C). To avoid the risk of incomplete reaction cycles and the physical condensation of precursors associated with low-temperature ALD (yielding films with defects and voids, and hence, degraded sub-bandgap optical properties, such as a reduced refractive index *n* and finite extinction coefficient *k*), an existing ALD process which uses TDMAH and H_2_O precursors and operates at *T*_p_ = 200 °C is modified (Fig. 1b) by (1) decreasing the process temperature to *T*_p_ = 95 °C; (2) increasing the TDMAH pulsing time, *t*_1_, from 0.25 to 1 s to enable a complete reaction with the OH monolayer resulting from the previous cycle; and (3) increasing the N_2_ purging times, *t*_2_ and *t*_4_, from 12 to 75 s to ensure full removal of the excessive precursors and reaction byproducts (see “Materials and methods”). As revealed by X-ray diffraction characterization (Supplementary Information, Section II) and spectroscopic ellipsometry measurements (Fig. 1c and Supplementary Information, Section III), HfO_2_ films deposited using the modified low-temperature ALD process are amorphous and characterized by a high refractive index (*n* > 2.1) and negligible optical loss (*k* ≈ 0) over a UV wavelength interval 220 nm ≤ *λ*_0_ ≤ 380 nm spanning the full mid-UV and near-UV ranges and more than half of the deep-UV range. The measured wavelength dependences of *n* and *k* closely match those of a film grown using the 200 °C reference ALD process (Supplementary Information, Fig. S4), demonstrating that the optical quality of the deposited HfO_2_ can be maintained at significantly lower ALD process temperatures with a suitable Hf precursor and a proper adjustment of the pulsing and purging times. Note that the 95 °C-ALD-deposited HfO_2_ films exhibit a high refractive index (*n* > 2.0) and zero optical loss (*k* = 0) in the visible range (380 nm ≤ *λ*_0_ ≤ 800 nm), making the films suitable for the fabrication of low-loss metasurface devices in this wavelength range as well (Supplementary Information, Fig. S5).

Using ZEP resist for e-beam lithography and the low-temperature TDMAH-based ALD process for the HfO_2_ deposition, the proposed Damascene fabrication process is applied to yield defect-free metasurfaces, each consisting of a large array of densely packed HfO_2_ nanopillars on a UV-grade fused silica substrate (Fig. 1f). The nanopillars have uniform heights, circular (Fig. 1d), or elliptical (Fig. 1e) in-plane cross sections, and are characterized by straight, vertical, and smooth sidewalls (Fig. 1d, e, and Supplementary Information, Figs. S7–S11). The nanopillar rotation angle and two principle axis lengths in the plane of the metasurfaces (*θ*, *D*_1_, and *D*_2_, respectively, where *θ* = 0 and *D*_1_ = *D*_2_ = *D* in the case of a circular cross-section) vary as a function of the nanopillar position (with 0 ≤ *θ* < *π* and 50 nm ≤ (*D*_1_, *D*_2_) ≤ 160 nm) depending on the optical function implemented by the metasurface. The nanopillar height *H* varies depending on the operation wavelength of the metasurface (400 nm ≤ *H* ≤ 550 nm).

We first demonstrate lenses, self-accelerating beam generators, and holograms based on polarization-independent metasurfaces consisting of nanopillars with in-plane circular cross-sections, that operate at near-UV wavelengths of 364 and 325 nm (corresponding to emission lines of argon-ion and helium–cadmium lasers, respectively) with efficiencies up to 72%. Further exploiting the high patterning fidelity of the Damascene technique and leveraging the negligible optical loss of the as-deposited HfO_2_ dielectric material across most of the UV regime, we scale down the metasurface critical dimensions to realize polarization-independent holograms operating at a deep-UV wavelength of 266 nm (corresponding to the emission line of an optical parametric oscillator pumped by a nanosecond Q-switched Nd:YAG laser), with relatively high efficiencies (>60%). Finally, by opening up the design space with the three degrees of freedom provided by elliptically shaped nanopillars (*θ*, *D*_1_, and *D*_2_), compared to the single degree of freedom provided by circularly shaped nanopillars (*D*), we realize spin-multiplexed metasurfaces that impart independent phase shift profiles onto light emerging from the device under illumination with left-handed circularly polarized (LCP) or right-handed circularly polarized (RCP) light. The implemented self-accelerating beam generators and spin-multiplexed metaholograms operate at UV wavelengths of 364 and 266 nm, respectively, with efficiencies up to 61%.

### Polarization-independent UV metasurfaces

Each polarization-independent metasurface implemented in this study (lens, self-accelerating beam generator, and hologram) consists of a square lattice of HfO_2_ cylindrical nanopillars, where the diameter of each pillar varies as a function of its position within the lattice. Each nanopillar acts as a truncated dielectric waveguide with top and bottom interfaces of low reflectivity, through which light propagates with a transmittance and phase shift controlled by the pillar height *H*, pillar diameter *D*, and lattice spacing *P*. For each targeted operation wavelength (*λ*_0_ = 364, 325, and 266 nm), a corresponding pillar height (*H* = 550, 500, and 400 nm, respectively) and subwavelength lattice spacing (*P* = 200, 190, and 150 nm, respectively) are chosen, along with a range of pillar diameters that yield phase shifts varying over a full range of 2*π*, while maintaining a relatively high and constant transmittance ([50, 160 nm], [50, 150 nm], and [50, 110 nm], respectively). The detailed design procedure is elaborated in Supplementary Information, Section VIII.

**Fig. 2:**
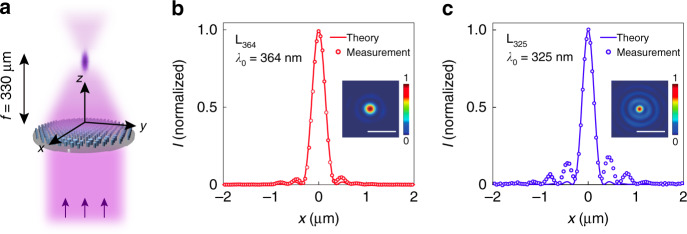
Polarization-independent near-UV metalenses. **a** Schematic representation of focusing by a metalens, L_364_ or L_325_, under normal-incidence, plane-wave illumination at *λ*_0_ = 364 or 325 nm, respectively. **b**, **c** Cross-focus cuts and intensity distributions in the focal plane, as measured for metalenses L_364_ and L_325_, respectively. The theoretically predicted cross-focus cuts are plotted for reference. Scale bars: 1 µm

As a first demonstration of polarization-independent UV metasurfaces, two 500-µm-diameter, polarization-independent metalens designs, L_364_ and L_325_, with an identical numerical aperture of NA = 0.6 (corresponding to a focal length of *f* = 330 μm), are implemented to focus UV light at respective free-space wavelengths of *λ*_0_ = 364 and 325 nm (Fig. 2a). Singlet-mode focusing of a plane wave can be achieved by implementing the radially symmetric phase shift function $\varphi ^{\mathrm {L}}( {x,y,\lambda _0}) = {\mathrm {mod}}( {( {2\pi /\lambda _0})\,( {f - \sqrt {x^2 + y^2 + f^2} }),2\pi }),$ where *f* is the focal distance normal to the plane of the lens (along the *z* direction), *x* and *y* are in-plane distances along orthogonal directions from the center of the lens, and normal incidence is assumed. Each measured intensity distribution at the metalens focal plane (Supplementary Information, Section IX) reveals a circularly symmetric focal spot, characterized by a cross-section that closely matches the intensity distribution theoretically predicted for a diffraction-limited lens with a numerical aperture of NA = 0.6 and given by the Airy disk function $I\left( x \right) = \left[ {2J_1(A)/A} \right]^2$, where *J*_1_ is the Bessel function of the first kind of order one, and $A = 2\pi {\mathrm{NA}}x/\lambda _0$ (Fig. 2b, c). Metalens L_325_ exhibits a less-than-ideal focusing profile with larger side lobes, which could be due to fabrication imperfections and a nonideal realization of the required phase shift profile. The focusing efficiencies, defined as the ratio of the optical power of the focused spot to the total power illuminating the metalens, are (55.17 ± 2.56)% (L_364_) and (56.28 ± 1.37)% (L_325_). The cited uncertainties represent one standard deviation of the measured data.

**Fig. 3:**
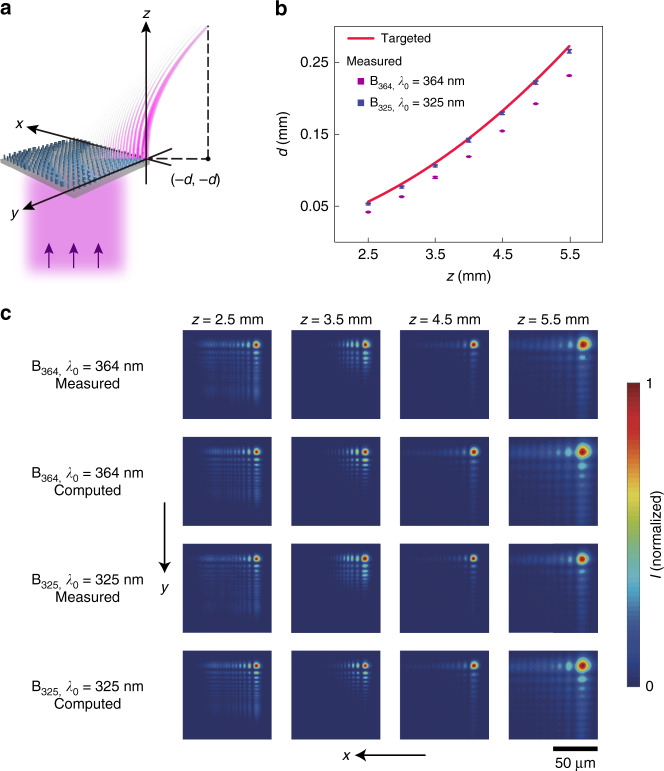
Polarization-independent near-UV self-accelerating beam generators. **a** Schematic representation of the generation of a self-accelerating beam by a metasurface, B_364_ or B_325_, under normal-incidence, plane-wave illumination at *λ*_0_ = 364 or 325 nm, respectively. **b** Measured transverse deflection in different *z* planes (ranging from 2.5 to 5.5 mm, with an increment of 0.5 mm) for the illumination of B_364_ and B_325_, respectively, at respective operation wavelengths *λ*_0_ = 364 and 325 nm. The error bars denote one standard deviation of the measured data. The targeted beam trajectory, $d = 9z^2$, is shown for reference. **c** Measured and computed *xy*-plane intensity distributions (normalized) at different *z* planes for both devices at their designated wavelengths of operation. Each distribution is displayed over an equal square area with a side length of 120 µm but shifted along the −*xy* direction as a function of increasing *z*, such that the center of the main lobe maintains an invariant position within each image

Next, we demonstrate polarization-independent metasurfaces that can transform a normally incident plane wave into a diffraction-free output beam propagating along a curved trajectory, i.e., a self-accelerating beam (SAB)^31–33^. Two 270-µm-square SAB generator designs, B_364_ and B_325_, are implemented for operation at the respective wavelengths of 364 and 325 nm (Fig. 3a). The SAB generator design and operation are conveniently described using a Cartesian coordinate system in which the constituent metasurface is located in the *z* = 0 plane and the first *xy* quadrant, with one corner positioned at the origin. The implemented SAB for each targeted free-space wavelength, *λ*_0_ = 364 and 325 nm, is characterized by an L-shaped wave-packet of the main lobe centered on the trajectory $y = x = - az^2$, where *a* = 9 m^−1^ (in other words, originating from (0, 0, 0) and propagating in the +*z* direction in a curved trajectory confined to the plane *y* = *x* with a height above the surface given by $z = \sqrt {d/a}$, where $d = \left| x \right| = \left| y \right|$ is the lateral displacement). The targeted SAB can be generated by implementing a phase shift profile $\varphi ^{\mathrm {B}}\left( {x,y,\lambda _0} \right) = {\mathrm {mod}}( { - \frac{{8\pi }}{{3\lambda _0}}\sqrt a ( {x^{\frac{3}{2}} + y^{\frac{3}{2}}}),2\pi })$ in the metasurface^34^. The measured lateral displacement values *d*(*z*) are observed to closely match, in each case, the calculated values based on the targeted trajectory (Fig. 3b and Supplementary Information, Section X). The experimental SAB generated by each device exhibits diffraction-free characteristics with *xy*-plane intensity distributions similar to the intensity distributions numerically computed using the angular spectrum representation method^35^, assuming an ideal metasurface realization with both the designed phase shift profile *φ*^B^ and unity transmittance *T* (Fig. 3c). The measured efficiencies, defined as the ratio of the total optical power of the SAB in the *z* = 5 mm plane to the total power illuminating the metasurface, are (46.75 ± 2.31)% (B_364_) and (67.42 ± 4.43)% (B_325_). The efficiencies compare favorably to that of a recently reported TiO_2_-based self-accelerating beam generator operating at visible frequencies^36^.

**Fig. 4:**
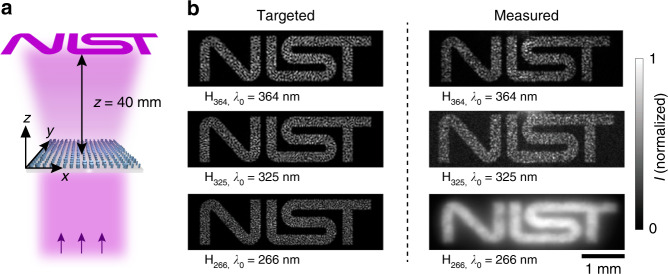
Polarization-independent near-UV and deep-UV metaholograms. **a** Schematic representation of the holographic image projection by a metahologram, H_364_, H_325_, or H_266_, under normal-incidence, plane-wave illumination at *λ*_0_ = 364, 325, or 266 nm, respectively. **b** Targeted (left panel) and measured (right panel) holographic images projected by the metaholograms H_364_, H_325_, and H_266_ in the *z* = 40 mm plane

As a final demonstration of polarization-independent UV metasurfaces, we demonstrate three metaholograms, denoted H_364_, H_325_, and H_266_, operating at three respective UV wavelengths *λ*_0_ = 364, 325, and 266 nm (Fig. 4a). Implementing computer-generated holograms with metasurfaces enables high-efficiency and low-noise operation, fine spatial resolution, a compact footprint, and multiplexing capability^37–40^. Each demonstrated metahologram, which occupies a square area with a side length of 270 µm, is mapped to a Cartesian coordinate system in which the constituent metasurface is located in the *z* = 0 plane and the first *xy* quadrant, with one corner positioned at the origin. The Gerchberg–Saxton algorithm^41^ is employed to calculate the phase shift profiles, $\varphi _{364}^{\mathrm {H}}(x,y,\lambda _0)$, $\varphi _{325}^{\mathrm {H}}(x,y,\lambda _0)$, and $\varphi _{266}^{\mathrm {H}}(x,y,\lambda _0)$, required to project a holographic “NIST” image located in the *z* = 40 mm plane, under normal-incidence, plane-wave illumination (Supplementary Information, Section XI). An additional offset of *y* = −3 mm is added to avoid overlap of the generated holographic image with the residual directly transmitted beam. The images projected by metaholograms H_364_, H_325_, and H_266_ are measured (Supplementary Information, Sections XII and XIII) and displayed in the right panel of Fig. 4b. Each of the experimental holographic images faithfully replicates the shape of the corresponding target image (left panel of Fig. 4b), numerically computed assuming an ideal metahologram realization with both the designed phase shift profile *φ*^H^ for a given operation wavelength and unity transmittance *T*. In addition, the speckle patterns filling the shapes of the measured images projected by metaholograms H_364_ and H_325_ exhibit numerous similarities with those of the corresponding target images; the as-measured holographic image projected by metahologram H_366_ does not offer the possibility of such a comparison due to the employed fluorescence transduction characterization scheme, which washes out the details of the speckle patterns. The measured efficiencies for metaholograms H_364_ and H_325_, defined as the ratio of the total optical power of the holographic image to the total power illuminating the structure, are (62.99 ± 4.14)% and (71.78 ± 2.06)%, respectively. The measured efficiency for metahologram H_266_, defined as the ratio of the total fluorescence power of the holographic image to the fluorescence power of the light illuminating the structure (Supplementary Information, Section XIII), is (60.67 ± 2.60)%. These efficiency values are comparable to those of recently reported TiO_2_-based metaholograms operating in the visible range^26^.

### Spin-multiplexed UV metasurfaces

Metasurfaces have been demonstrated to switch between distinct optical outputs, such as different holographic images or differently oriented beams, under the control of the fundamental optical state of the input beam, e.g., polarization^42,43^, or a spatial feature of the input beam such as the angle of incidence^44^. Here, we demonstrate, for the first time, spin-multiplexed UV metasurfaces that can switch between distinct outputs depending on the handedness of the input light (left-hand circularly polarized, LCP, or right-hand circularly polarized, RCP). The detailed design procedure is elaborated in Supplementary Information, Section XIV.

**Fig. 5:**
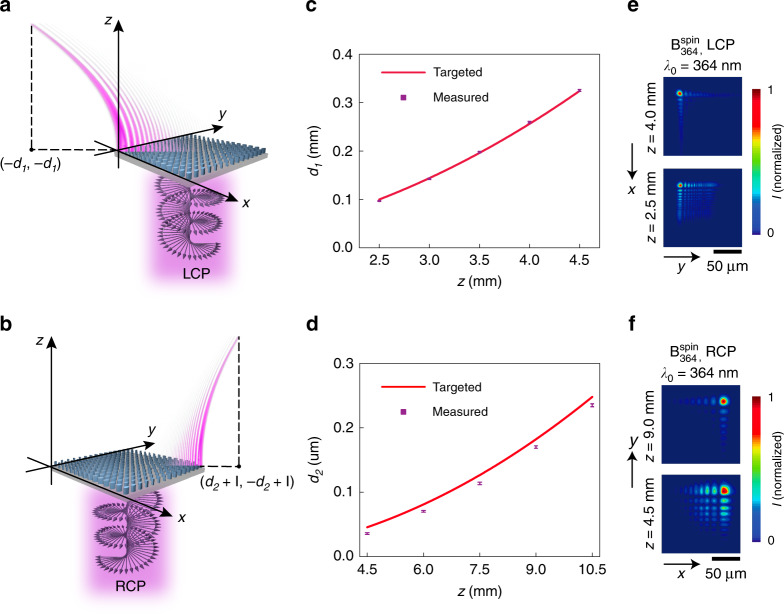
Spin-multiplexed near-UV self-accelerating beam generator. **a**, **b** Schematic representation of the generation of a self-accelerating beam by the spin-multiplexed metasurface, ${\mathrm{{B}}}_{364}^{{\mathrm{spin}}}$, under normal-incidence, plane-wave LCP **a** or RCP **b** illumination at *λ*_0_ = 364 nm. **c** Measured transverse deflection in different *z* planes (ranging from 2.5 to 4.5 mm, with an increment of 0.5 mm) for LCP illumination of ${\mathrm{{B}}}_{364}^{{\mathrm{spin}}}$ at its operation wavelength of *λ*_0_ = 364 nm. The error bars denote one standard deviation of the measured data. The targeted beam trajectory, $d = 16z^2$, is shown for reference. **d** Measured transverse deflection in different *z* planes (ranging from 4.5 to 10.5 mm, with an increment of 1.5 mm) for RCP illumination of ${\mathrm{B}}_{364}^{{\mathrm{spin}}}$ at its operation wavelength of *λ*_0_ = 364 nm. The error bars denote one standard deviation of the measured data. The targeted beam trajectory, $d = 2.25z^2$, is shown for reference. **e, f** Measured *xy*-plane intensity distributions (normalized) in different *z* planes for the device at its designated wavelength of operation under either LCP illumination **e** or RCP illumination **f**. Each distribution is displayed over an equal square area with a side length of 140 µm but shifted along the −*xy* **e** or *xy* **f** direction as a function of increasing *z*, such that the center of the main lobe maintains an invariant position within each image

As a first demonstration of a polarization-dependent, spin-multiplexed UV metasurface, we implement a self-accelerating beam generator operating at *λ*_0_ = 364 nm, denoted as ${\mathrm{{B}}}_{364}^{{\mathrm{spin}}}$, that generates SABs following different trajectories under the control of the handedness of circularly polarized incident light. The spin-multiplexed SAB generator, which occupies a square area with a side length of *l* = 330 µm, is referenced to a Cartesian coordinate system in which the constituent metasurface is located in the *z* = 0 plane and the first *xy* quadrant, with one corner positioned at the origin. Two distinct phase shift profiles, $\varphi ^{{\mathrm {LCP}}}\left( {x,y,\lambda _0} \right) = {\mathrm {mod}}\left( { - \frac{{8\pi }}{{3\lambda _0}}\sqrt {16} \left( {x^{\frac{3}{2}} + y^{\frac{3}{2}}} \right),\,2\pi } \right)$ and $\varphi ^{{\mathrm {RCP}}}\left( {x,y,\lambda _0} \right) = {\mathrm {mod}}\left( { - \frac{{8\pi }}{{3\lambda _0}}\sqrt {2.25} \left( {\left( {l - x} \right)^{\frac{3}{2}} \;+\; \left( {l - y} \right)^{\frac{3}{2}}} \right),\,2\pi } \right)$, are targeted for the device operation to yield SABs exiting the metasurface from opposite corners and following different trajectories, $y = x = - d_1 = - 16z^2$ and $\left( {y - l} \right) = \left( {x - l} \right) = d_2 = 2.25z^2$, under LCP and RCP illuminations, respectively (Fig. 5a, d). The measured lateral displacement values, *d*_1_(*z*) and *d*_2_(*z*), are observed to closely match, in each case, the calculated values based on the targeted trajectory (Fig. 5c, d). The experimental SAB generated by the device exhibits diffraction-free characteristics with *xy*-plane intensity distributions (Fig. 5e, f) similar to the targeted intensity distributions (Supplementary Information, Figs. S14 and S15), numerically computed assuming an ideal metasurface realization with both the designed phase shift profile *φ*^LCP^ (*φ*^RCP^) and unity transmittance *T*. The measured efficiency under LCP illumination (RCP illumination), defined as the ratio of the total optical power of the SAB in the *z* = 4.5 mm [*z* = 10.5 mm] plane to the total power illuminating the metasurface, is (38.42 ± 1.95)% [(61.90 ± 2.03)%]. The reduced efficiency under LCP illumination, compared to the RCP case, can be attributed to challenges associated with implementing a phase shift profile of a higher spatial gradient (Supplementary Information, Section XVI).

**Fig. 6:**
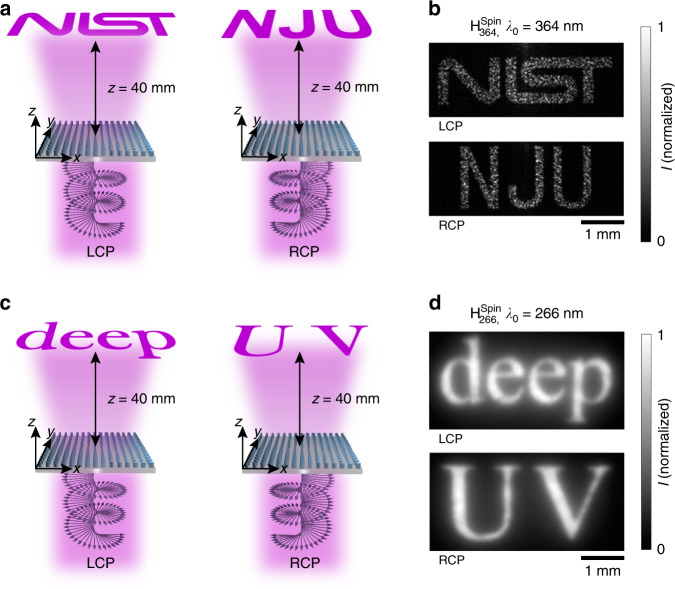
Spin-multiplexed near- and deep-UV metaholograms. **a** Schematic representation of the holographic image projection by the spin-multiplexed metahologram ${\mathrm{H}}_{364}^{{\mathrm{spin}}}$ under LCP or RCP illumination at *λ*_0_ = 364 nm. **b** Measured holographic images projected by metahologram ${\mathrm{H}}_{364}^{{\mathrm{spin}}}$ in the *z* = 40 mm plane under LCP illumination (top image) and RCP illumination (bottom image). **c** Schematic representation of the holographic image projection by the spin-multiplexed metahologram ${\mathrm{H}}_{266}^{{\mathrm{spin}}}$ under LCP or RCP illumination at *λ*_0_ = 266 nm. **d** Measured holographic images projected by metahologram ${\mathrm{H}}_{266}^{{\mathrm{spin}}}$ in the *z* = 40 mm plane under LCP illumination (top image) and RCP illumination (bottom image)

Next, we demonstrate a spin-controlled metahologram operating at the same near-UV wavelength of 364 nm. The 330-µm-square metahologram, ${\mathrm{H}}_{364}^{{\mathrm{spin}}}$, located in the *z* = 0 plane, is designed to project a holographic “NIST” image (for LCP illuminating light) and “NJU” image (for RCP illuminating light) at *λ*_0_ = 364 nm, all located in the *xy*-plane at *z* = 40 mm with an offset of *y* = −3 mm (Fig. 6a; the corresponding phase shift profiles are plotted in the Supplementary Information, Fig. S17). Both of the experimentally captured holographic images (Fig. 6b) faithfully replicate the shape of the corresponding targeted image computed from the designed phase profiles, including some fine grain details (Supplementary Information, Fig. S18). The measured efficiencies, defined as the ratio of the total optical power of the holographic image to the total power illuminating the metahologram, are (54.02 ± 2.22)% (under LCP illumination) and (53.76 ± 2.42)% (under RCP illumination), respectively.

Finally, a spin-multiplexed metahologram, ${\mathrm{H}}_{266}^{{\mathrm{spin}}}$, occupying a square area with a side length of 320 µm, is implemented for operation at the deep-UV wavelength of 266 nm. The device, located in the *z* = 0 plane, is designed to project, at *λ*_0_ = 266 nm, a holographic “deep” image for LCP illumination and a holographic “UV” image for RCP illumination, where both images are located in the *z* = 40 mm plane with a lateral offset of *y* = −3 mm (Fig. 6c; the corresponding phase shift profiles are plotted in the Supplementary Information, Fig. S19). Each of the experimental holographic images (Fig. 6d) faithfully replicates the shape of the corresponding target image (Supplementary Information, Fig. S20), including subtle details of the chosen font, such as the linewidth variation and serif. The measured efficiencies, defined as the ratio of the total fluorescence power of the holographic image to the fluorescence power of light illuminating the structure, are (58.95 ± 1.95)% under LCP illumination and (61.23 ± 1.49)% under RCP illumination.

## Discussion

Given the negligible extinction coefficient of the low-temperature-ALD deposited HfO_2_ down to its bandgap (*λ*_0_ ≈ 217 nm) and the high patterning fidelity of the Damascene process, it should be straightforward to push the metasurface operation wavelengths to significantly shorter values than those demonstrated here. In addition, an experimental demonstration of a broader range of device functionalities in the deep-UV regime other than hologram projection should be possible by using a continuous-wave light source and an appropriate direct imaging system. Moreover, the efficiency of HfO_2_-based metasurface devices can be improved by further optimizing the Damascene process or by employing advanced metasurface design strategies, such as topology optimization^45^ and the generalized Huygens principle^46,47^.

In conclusion, an assortment of high-performance metasurface components operating in the UV regime, including wavelengths down to the record-short deep-UV range, is demonstrated by using HfO_2_, a CMOS-compatible, wide-bandgap, and low-loss dielectric material, and an associated fabrication process based on low-temperature ALD and Damascene lithography. This approach paves the way towards further development of “flat” UV optical elements with customized functionalities and their integration into chip-scale nanophotonic systems, enabling applications such as atom trapping, fluorescence imaging, and circular dichroism spectroscopy with a compact form factor.

## Materials and methods

### Metasurface fabrication process

As the first step in the metasurface fabrication process, 500-µm-thick, double-side-polished UV-grade fused silica wafers are vapor-coated (150 °C) with an adhesion-enhancing monolayer of hexamethyldisilizane (HMDS). A layer of ZEP 520A resist is spin-coated onto the substrate, followed by baking on a hot plate at 180 °C for 10 min. The spin speed is adjusted to yield a resist thickness varying between 400 and 550 nm (as characterized by spectroscopic ellipsometry), depending on the specific metasurface design. To suppress charging during electron beam (e-beam) lithography, a 20-nm-thick Al layer is thermally evaporated onto the ZEP layer (deposition rate of 0.1 nm/s). The ZEP-resist template is fabricated using e-beam lithography (accelerating voltage of 100 kV and beam current of 0.2 nA), followed by Al layer removal (AZ 400 K 1:3 developer for 2 min and DI water for 1 min) and resist development (hexyl acetate for 2 min and isopropyl alcohol for 30 s). The deposition of HfO_2_ (deposition rate: 0.11 nm/cycle) is then performed using the low-temperature ALD described below. For all of the processed structures, the deposition thickness is chosen to be 200 nm, which not only exceeds the largest radius (or the largest semi-minor axis length) of the circular (or elliptical) openings of the exposed resist patterns for all metasurface designs, providing complete filling of the patterns, but also provides a substantial over-coating of the resist, yielding a quasi-planar top surface (Supplementary Information, Section VI). Following the ALD, the HfO_2_ layer is back-etched to the resist top surface using argon (Ar) ion milling (HfO_2_ mill rate of ≈0.4 nm/s). During the Ar ion milling, a non-patterned, planar HfO_2_ sample of the same initial thickness is also back-etched, and its film thickness is periodically monitored by spectroscopic ellipsometry to ensure that a proper milling time is employed. Finally, the remaining resist is removed by soaking in a solvent, yielding circular or elliptical HfO_2_ posts with smooth and straight sidewall profiles (due to the resist templating process), heights varying from 400 to 550 nm (depending on the specific metasurface), and aspect ratios varying from ≈3 to ≈11.

### Low-temperature TDMAH-based HfO_2_ ALD

In step 1 of the ALD cycle, TDMAH vapor (Hf [(CH_3_)_2_N]_4_) is pulsed into the ALD chamber for a duration of *t*_1_ = 1 s, reacting with the dangling O–H bonds on the hafnium-coated surface to create a new solid monolayer of ${\mathrm{Hf}}\left[ {({\mathrm{CH}}_3)_2{\mathrm{N}}} \right]_2{\mathrm{O}}$ and generate the gas by-product (CH_3_)_2_NH (dimethylamine). In step 2, high-purity nitrogen (N_2_) gas is flowed for a duration of *t*_2_ = 75 s to fully remove any un-reacted TDMAH vapor and dimethylamine by-product from the chamber. In step 3, water vapor is pulsed into the chamber for a duration of *t*_3_ = 60 ms, reacting with the ${\mathrm{Hf}}[({\mathrm{CH}}_3)_2{\mathrm{N}}]_2{\mathrm{O}}$ to create a monolayer of HfO_2_ on the surface. Finally, in step 4, the excessive water vapor and the dimethylamine reaction by-product are completely removed from the chamber by N_2_ purging for a duration of *t*_4_ = 75 s.

## Supplementary information

Supplementary Information
