# Supplementary material for: Low-loss metasurface optics down to the deep ultraviolet region
Source: Light Sci Appl. 2020 Apr 9;9:55. doi: 10.1038/s41377-020-0287-y (PMC7142140; doi:10.1038/s41377-020-0287-y)
Supplement: Supplementary file 1 — Supplementary Information [file 41377_2020_287_MOESM1_ESM.pdf]

Supplementary Information for

Low-loss Metasurface Optics down to the Deep Ultraviolet Region

Cheng Zhang[1,2,3*], Shawn Divitt[2,3], Qingbin Fan[4], Wenqi Zhu[2,3], Amit Agrawal[2,3], Yanqing Lu[4], Ting Xu[4*] and Henri J. Lezec[2*]

[1.] School of Optical and Electronic Information & Wuhan National Laboratory for Optoelectronics, Huazhong University of Science and Technology, Wuhan, 430074, China.

[2.] Physical Measurement Laboratory, National Institute of Standards and Technology, Gaithersburg, MD, 20899, USA.

[3.] Maryland Nanocenter, University of Maryland, College Park, MD, 20742, USA.

[4.] National Laboratory of Solid State Microstructures, College of Engineering and Applied Sciences and Collaborative Innovation Center of Advanced Microstructures, Nanjing University, Nanjing, 210093, China.

These authors contributed equally: Cheng Zhang, Shawn Divitt and Qingbin Fan.

[*] Corresponding authors. Cheng Zhang, Email: cheng.zhang@hust.edu.cn; Ting Xu, Email: xuting@nju.edu.cn; Henri J. Lezec, Email: henri.lezec@nist.gov

This Supplementary Information includes:

Section I. Transmittance of the UV-grade fused silica substrate

Section II. X-ray diffraction (XRD) characterization of ALD-deposited HfO_2

Section III. Spectroscopic ellipsometry characterization of low-temperature ALD-deposited HfO_2

Section IV. Comparison of UV optical properties of ALD-deposited HfO_2 at 95 °C and 200 °C

Section V. Optical properties of ALD-deposited HfO_2 and TiO_2 over the UV and visible range

Section VI. Atomic force microscopy (AFM) characterization of the top surfaces of the patterned and non-patterned resist areas after the ALD coating

Section VII. Scanning electron micrographs of UV metasurfaces

Section VIII. Design of the polarization-independent metasurfaces

Section IX. Characterization procedure for metalenses L_{364} and L_{325}

Section X. Characterization procedure for self-accelerating beam generators B_{364} and B_{325}

Section XI. Designed phase shift profiles φ_{364}^{H}, φ_{325}^{H}, and φ_{266}^{H} for metaholograms H_{364}, H_{325}, and H_{266}

Section XII. Characterization procedure for metaholograms H_{364} and H_{325}

Section XIII. Characterization procedure for metahologram H_{266}

Section XIV. Design of spin-multiplexed metasurfaces

Section XV. Characterization procedure for spin-multiplexed self-accelerating beam generator $\mathrm{B}_{364}^{\mathrm{spin}}$

Section XVI. Discussion of reduced device efficiency under LCP illumination for $\mathrm{B}_{364}^{\mathrm{spin}}$

Section XVII. Implementation of spin-multiplexed metahologram $\mathrm{H}_{364}^{\mathrm{spin}}$

Section XVIII. Implementation of spin-multiplexed metahologram $\mathrm{H}_{266}^{\mathrm{spin}}$

I. Transmittance of the UV-grade fused silica substrate

As displayed in Fig. S1, the 500-μm-thick, UV-grade fused silica wafer utilized as metasurface substrate provides a high optical transmittance (> 90 %) over the wavelength range exploited in this study (266 nm to 364 nm).

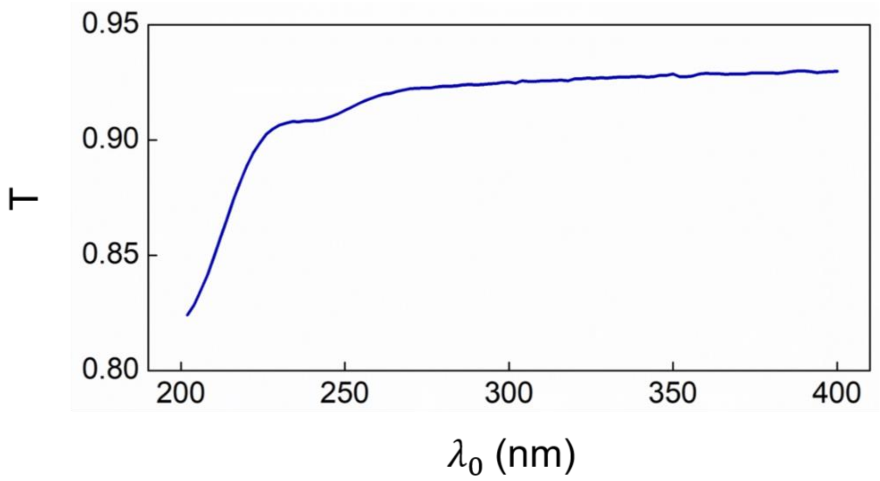

Fig. S1. Measured transmittance versus wavelength of a 500-μm-thick, UV-grade fused silica wafer.

II. X-ray diffraction (XRD) characterization of ALD-deposited HfO_2

Glancing angle X-ray diffraction (XRD) characterization is performed on a 200-nm-thick HfO_2 film grown by the low-temperature ALD on an amorphous UV-grade fused silica substrate, using a theta-2theta scan configuration. The angle of incidence is 0.7°, the scan range is from 30° to 120°, and the scan speed is set to 2°/min. The XRD angular spectrum (Fig. S2) reveals that the ALD-deposited HfO_2 is amorphous as evidenced by the absence of diffraction peaks.

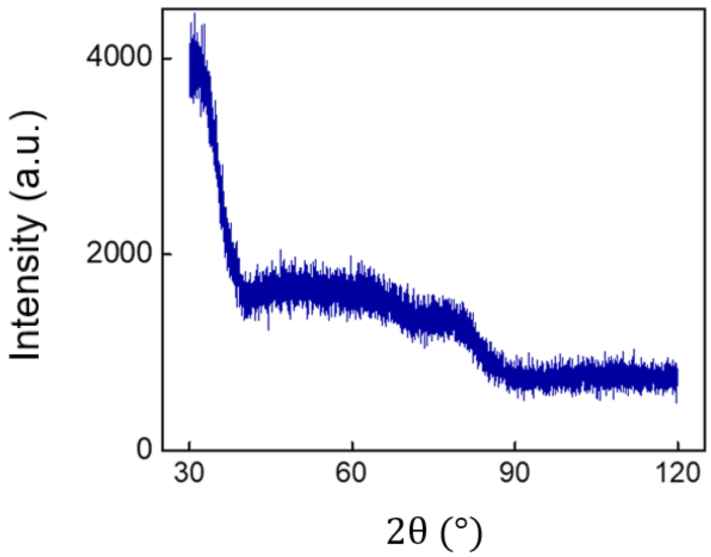

Fig. S2. X-ray diffraction (XRD) angular spectrum of ALD-deposited HfO_2.

III. Spectroscopic ellipsometry characterization of low-temperature ALD-deposited HfO_2

A 200-nm-thick HfO_2 film is grown by low-temperature ALD on a silicon wafer coated with a 300-nm-thick thermal oxide layer. The film's optical properties are characterized by reflection-mode spectroscopic ellipsometry using the interference enhancement method[1, 2], at three different angles of incidence (55°, 65°, and 75°) with respect to the normal to the plane of the HfO_2 layer. The dielectric function of the HfO_2 is modelled by a Tauc-Lorentz oscillator. The measured and best-match modeled Psi (Ψ) and Delta (Δ) curves (Fig. S3) display close correspondence, as evidenced by a low mean-squared-error for the fit (MSE = 6.686). The corresponding curves for the extracted values of refractive index n and extinction coefficient k are plotted in the main text (Fig. 1c).

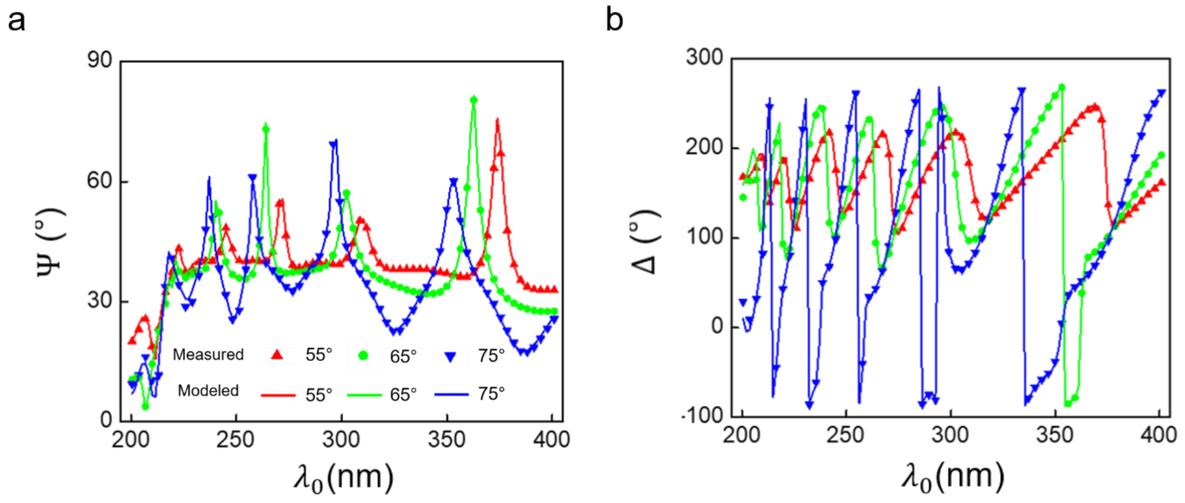

Fig. S3. Measured and best-match modeled Psi and Delta curves for ellipsometric characterization of low-temperature ALD-deposited HfO_2. The legend in Fig. S3a applies to Fig. S3b.

IV. Comparison of UV optical properties of ALD-deposited HfO_2 at 95 °C and 200 °C

The UV optical properties (characterized by spectroscopic ellipsometry) of HfO_2 deposited by TDMAH / water-based ALD using the new, low-temperature process (process temperature: T_p = 95 °C, TDMAH pulsing time: t_1 = 1 s, N_2 purging time: t_2 = 75 s, H_2O pulsing time: t_3 = 60 ms, N_2 purging time: t_4 = 75 s, and deposition rate: 0.110 nm/cycle) are compared to those resulting from deposition using a standard process (process temperature: T_p = 200 °C, TDMAH pulsing time: t_1 = 250 ms, N_2 purging time: t_2 = 12 s, H_2O pulsing time: t_3 = 60 ms, N_2 purging time: t_4 = 12 s, and deposition rate: 0.106 nm / cycle). Over the UV range, the two films exhibit (Fig. S4) virtually identical extinction coefficients k (where $k \approx 0$ for $\lambda_0 \geq 220$ nm), and refractive indices n that differ only slightly (where values for the film deposited at 95 °C are at most lower by ≈ 0.03 than those for film deposited at 200 °C, at any given wavelength in that range).

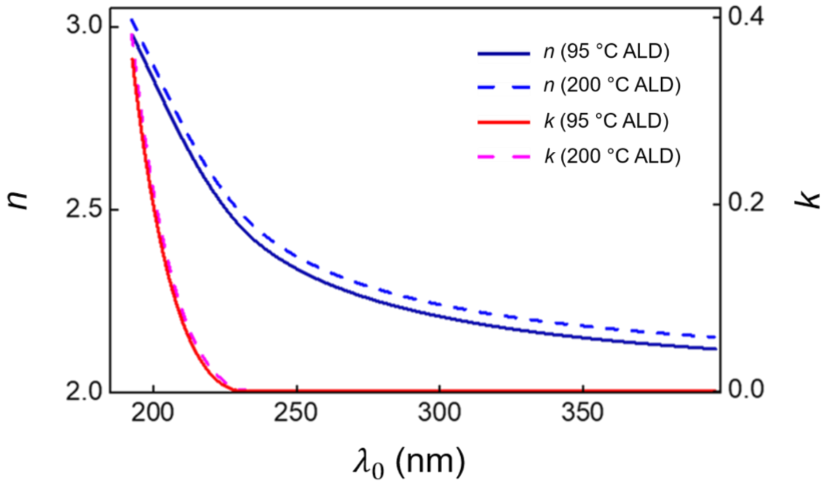

Fig. S4. Measured refractive index n and extinction coefficient k of HfO_2 films deposited by ALD at 95 °C and 200 °C, respectively.

V. Optical properties of ALD-deposited HfO_2 and TiO_2 over the UV and visible range

As displayed in Fig. S5, the 95°C-ALD-deposited HfO_2 film exhibits a high refractive index ($n > 2.0$) and zero optical loss ($k = 0$) in the visible range (380 nm $\leq \lambda_0 \leq$ 800 nm), making it suitable for fabricating low-loss metasurface devices in this wavelength range as well. The optical constants of a more commonly used material in this spectral range, TiO_2, is also shown for comparison.

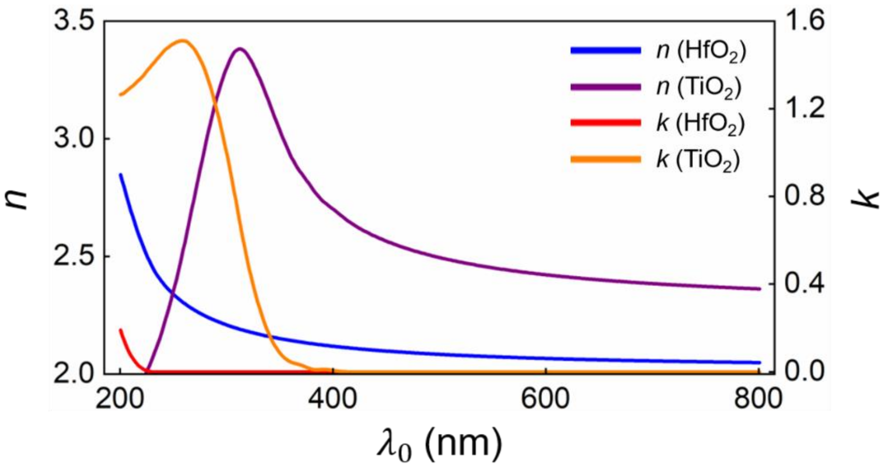

Fig. S5. Measured refractive index n and extinction coefficient k of the 95°C-ALD-deposited HfO_2 films and the 90°C-ALD-deposited TiO_2 films in the UV and visible range.

VI. Atomic force microscopy (AFM) characterization of the top surfaces of the patterned and non-patterned resist areas after the ALD coating

Atomic force microscopy (AFM) characterization is performed on the top surfaces of a patterned resist area (Fig. S6, Left) and a non-patterned resist area (Fig. S6, Right) after the 200-nm-thick HfO_2 ALD coating. Both the patterned and non-patterned areas exhibit similar quasi-planar surface morphologies with RMS roughness values of 4.32 nm and 4.13 nm, respectively.

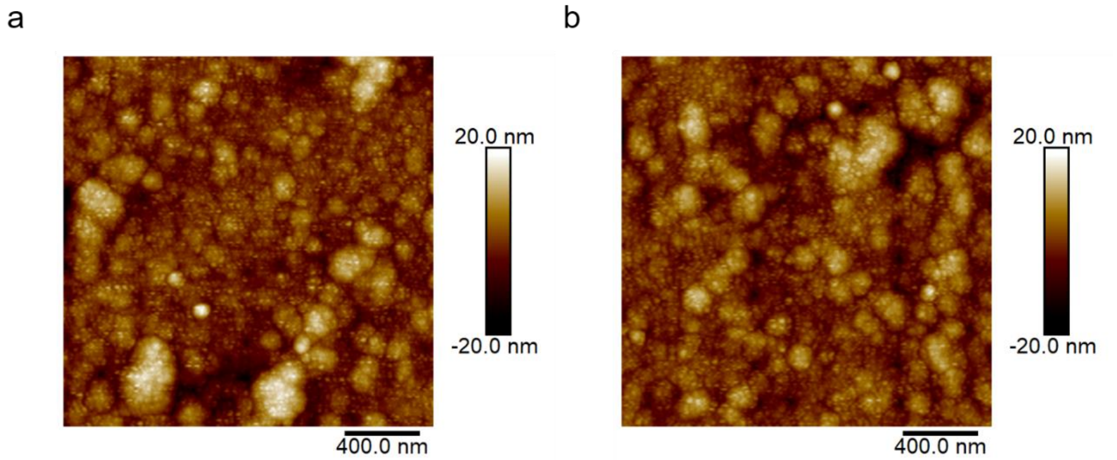

Fig. S6. Atomic force microscopy (AFM) characterization over the top surfaces of a patterned resist area (left) and non-patterned resist area (right) after the 200-nm-thick HfO_2 ALD coating. The scan is performed over a square area with a side length of 2 μm.

VII. Scanning electron micrographs of UV metasurfaces

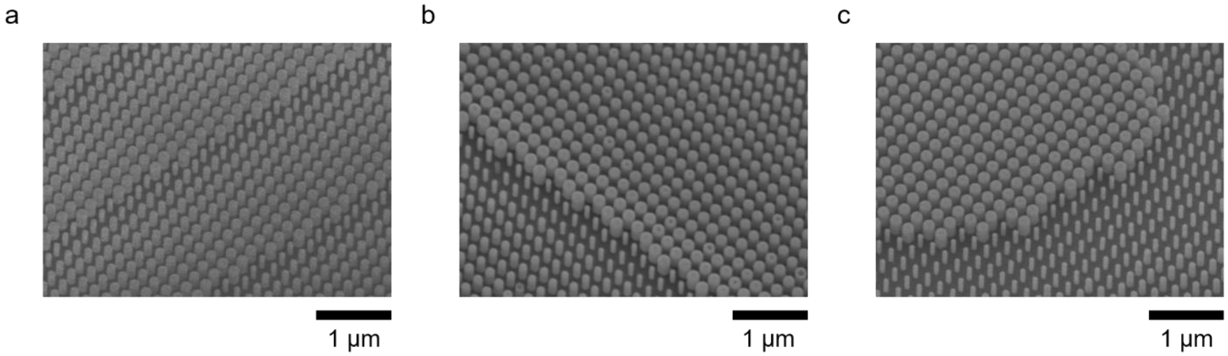

Fig. S7. Scanning electron micrographs (viewing angle: 20°) of selected areas of polarization-independent metasurfaces designed for operation at λ_0 = 364 nm. **a**, Metalens L_{364} . **b**, Self-accelerating beam generator B_{364}. **c**, Metahologram H_{364}.

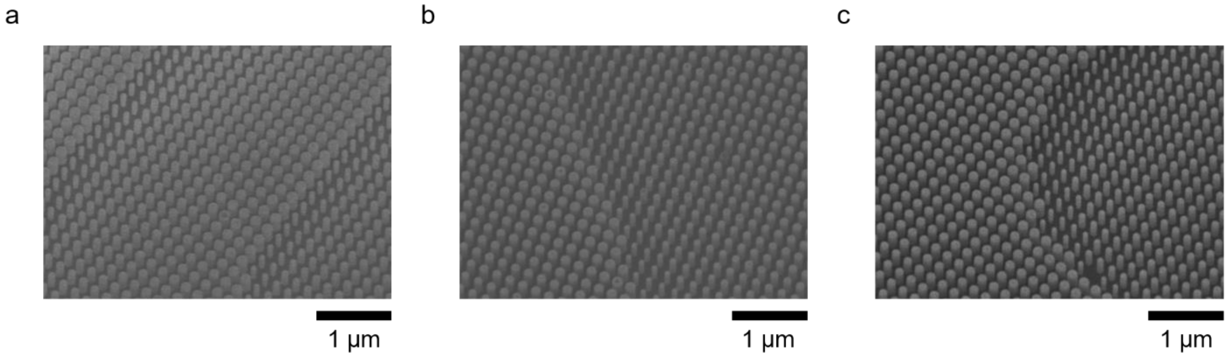

Fig. S8. Scanning electron micrographs (viewing angle: 20°) of selected areas of polarization-independent metasurfaces designed for operation at λ_0 = 325 nm. **a**, Metalens L_{325} . **b**, Self-accelerating beam generator B_{325}. **c**, Metahologram H_{325}.

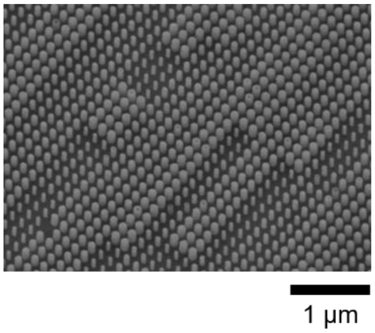

Fig. S9. Scanning electron micrograph (viewing angle: 20°) of selected areas of polarization-independent metahologram designed for operation at λ_0 = 266 nm, H_{266}.

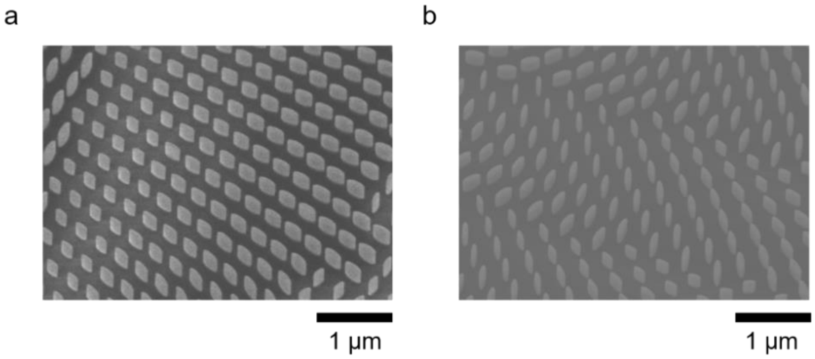

Fig. S10. Scanning electron micrograph (viewing angle: 20°) of selected areas of spin-multiplexed metasurfaces designed for operation at λ_0 = 364 nm. **a**, Metahologram $\mathrm{H}_{364}^{\mathrm{spin}}$. **b**, Self-accelerating beam generator $\mathrm{B}_{364}^{\mathrm{spin}}$.

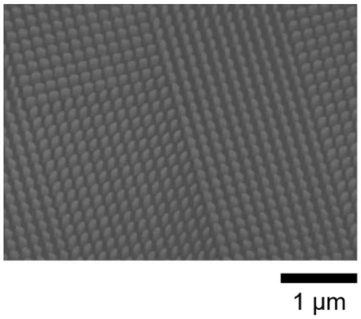

Fig. S11. Scanning electron micrograph (viewing angle: 20°) of selected areas of spin-multiplexed metahologram designed for operation at λ_0 = 266 nm, $\mathrm{H}_{266}^{\mathrm{spin}}$.

VIII. Design of the polarization-independent metasurfaces

The transmittance, T, and induced phase shift, φ, of an array of cylindrical pillars of diameter D, height H, and lattice spacing P, under plane-wave normal illumination at wavelengths λ_0 = 364 nm, 325 nm, and 266 nm, respectively, are computed using the finite-difference-time-domain (FDTD) simulations with periodic boundary conditions. Given a constant lattice spacing P over the entire metasurface, the function of the cylindrical pillars is to provide a relative propagation phase shift of up to $\varphi_{max} - \varphi_{min}$ = 2π, between the phase φ_{min} induced by the smallest-diameter pillar array (having a small filling factor compared to P, yielding a through-array propagation index $n_{min} \approx 1$, close to the refractive index of air) and the phase φ_{max} induced by the largest-diameter pillar array (having a larger filling factor compared to P, yielding a through-array propagation index $n_{max} \approx 2.1$, close to averaged index of refraction n of HfO_2 in the UV regime). This requirement sets a lower limit on possible values of H of $H_{min} = \lambda_0 / (n_{max} - n_{min}) \approx \lambda_0$.

Other constraints help to limit the extent of parameter space necessary to explore during the metasurface design process: (1) to avoid diffraction of transmitted light, the upper limit of P, P_{max}, is chosen such that $P_{max} \leq \lambda_0$; (2) to stay within the process tolerance of the Damascene lithography, the minimum value of D, D_{min}, is chosen such that D_{min} = 50 nm; (3) to maintain the mechanical stability of the resist template, the maximum possible value of D is chosen as ($P - 40$ nm). For each targeted free-space operation wavelength λ_0, different combinations of H, P are surveyed subject to the above requirements, along with the additional constraints that: (1) $\varphi(D) - \varphi(D_{min})$ span at least the full range of [0 2π], as D increases between D_{min} and a value $D_{max} \leq$ (P – 40 nm), where $\varphi(D_{max})$ = 2π, and (2) the transmittance T maintains a high and relatively constant value as D varies over the same range. Sets (H, P, D_{min}, and D_{max}) satisfying all those conditions are obtained for each of the targeted free-space operation wavelength, λ_0 = 364 nm, 325 nm, and 266 nm, of (550, 200, 50, 160), (500, 190, 50, 150), and (400, 150, 50, 110), respectively, where all values are expressed in nanometers (Fig. S12).

For each implemented polarization-independent metasurface device, the pillar height H and lattice spacing P are chosen based on the metasurface operating wavelength λ_0. The diameter of each

nano-cylinder over the metasurface plane is chosen such that the induced phase-shift $\varphi(D, x_c, y_c) = mod(\varphi^{PI}(x_c, y_c), 2\pi)$, where φ^{PI} is the required phase-shift profile for each metasurface, D is the diameter of the nanopillar, and (x_c, y_c) is the center position of each cylinder. For all polarization-independent metasurface designs, 32 discretized phase levels are chosen for φ^{PI}.

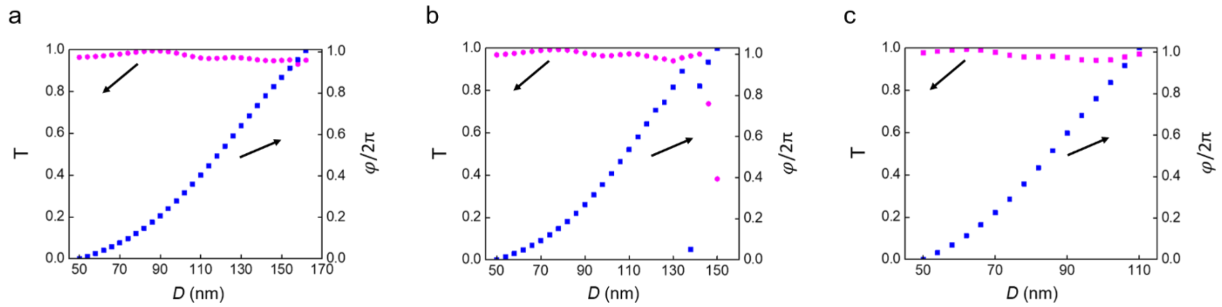

Fig. S12. Transmission intensity T and phase shift φ for an incident light of free-space wavelength λ_0 = 364 nm (a), 325 nm (b), and 266 nm (c), as a function of cylinder diameter *D*. For each free-space wavelength design (λ_0 = 364 nm, 325 nm, and 266 nm), a corresponding cylinder height (*H* = 550 nm, 500 nm, and 400 nm, respectively), sub-wavelength lattice spacing (*P* = 200 nm, 190 nm, and 150 nm, respectively), and maximum value of pillar diameter (D_{max} = 160 nm, 150 nm, and 110 nm, respectively) are chosen. For ease of display, the phase shift for pillar arrays with diameter *D* = 50 nm is set to zero for each design.

IX. Characterization procedure for metalenses $\mathbf{L_{364}}$ and $\mathbf{L_{325}}$

A continuous wave (CW) laser beam (diameter: ≈ 5 mm) illuminates either metalens L_{364} (using wavelength λ_0 = 364 nm) or L_{325} (using wavelength λ_0 = 325 nm) at normal incidence, yielding a focused spot in the focal plane of the metalens. The intensity distribution of the image in this plane is captured using a custom-built imaging system including an NA = 0.75 objective and an EMCCD camera. The magnification of the system, characterized at each wavelength by translating the focal spot within the field of view of the objective using a calibrated stage, is measured to be 420 and 617 for λ_0 = 364 and 325 nm, respectively. The physical size of the focal spot projected by metalenses L_{364} and L_{325} is then derived based on the CCD pixel size and magnification calibrated for respective operating wavelengths.

X. Characterization procedure for self-accelerating beam generators $\mathbf{B_{364}}$ and $\mathbf{B_{325}}$

A continuous wave (CW) laser beam (diameter: $\approx$ 5 mm) illuminates either self-accelerating beam generator B_{364} (using wavelength λ_0 = 364 nm) or B_{325} (using wavelength λ_0 = 325 nm) at the normal incidence. The intensity distributions of the generated self-accelerating beams in selected z-planes beyond the metasurface are recorded using custom-built imaging system including an NA = 0.75 objective and an EMCCD camera. Specific z-planes are addressed using a stage which translates the metasurface relative to the camera, along the direction of the laser beam. A 500-µm-diameter aperture is placed behind each metasurface substrate to completely separate the generated self-accelerating beam from directly transmitted light leaking around the edges of the metasurface (which occupies a square area with a side length of 270 µm). The physical size of the captured intensity profile is determined by comparison to the image of the area occupied by the metasurface.

XI. Designed phase shift profiles for metaholograms $\mathbf{H_{364}}$, $\mathbf{H_{325}}$, and $\mathbf{H_{266}}$

The Gerchberg-Saxton algorithm[4] is employed to calculate the respective metasurface phase shift profiles, $\varphi_{364}^{H}(x, y, \lambda_0)$, $\varphi_{325}^{H}(x, y, \lambda_0)$, and $\varphi_{266}^{H}(x, y, \lambda_0)$, required to produce a holographic "NIST" image at three different operation wavelengths λ_0 = 364 nm, 325 nm, and 266 nm (shown in Fig. S13 and implemented, respectively, by metaholograms $\mathrm{H_{364}}$, $\mathrm{H_{325}}$, and $\mathrm{H_{266}}$).

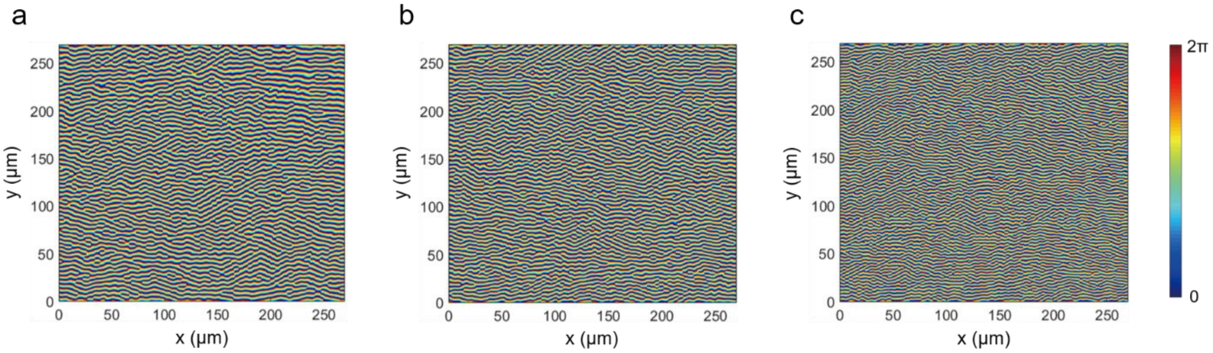

Fig. S13. Metasurface phase shift profiles, $\varphi_{364}^{H}(x, y, \lambda_0)$, $\varphi_{325}^{H}(x, y, \lambda_0)$, and $\varphi_{266}^{H}(x, y, \lambda_0)$, designed to produce a "NIST" holographic image for normal-incidence, plane-wave illumination at λ_0 = (a) 364 nm, (b) 325 nm, and (c) 266 nm (implemented by polarization independent metaholograms, $\mathrm{H_{364}}$, $\mathrm{H_{325}}$, and $\mathrm{H_{266}}$, respectively).

XII. Characterization procedure for metaholograms $\mathbf{H_{364}}$ and $\mathbf{H_{325}}$

A continuous wave (CW) laser beam (diameter: ≈ 5 mm) illuminates either metahologram H_{364} (using wavelength λ_0 = 364 nm) or H_{364} (using wavelength λ_0 = 325 nm) at normal incidence. An EMCCD camera is placed in the hologram formation plane located 40 mm beyond the metahologram, to directly record the projected holographic image. A 500-µm-diameter aperture is placed behind the metasurface substrate to completely separate the generated holographic image (projected with a 3-mm lateral offset) from directly transmitted light leaking around the edges of the metasurface (which occupies a square area with a side length of 270 µm).

XIII. Characterization procedure for metahologram $\mathbf{H_{266}}$

A normally incident, 266-nm pulsed laser (pulse duration: ≈ 5 ns, repetition rate: 10 Hz, beam diameter: ≈ 6 mm) is used to directly illuminate the polarization-independent metahologram H_{266}. A piece of fluorescent white paper is placed in the hologram formation plane located 40 mm beyond each metahologram and imaged with a custom-built imaging system including a lens and a CCD camera. Holographic images are recorded for device H_{266} under linearly-polarized illumination. 500 images each are recorded and averaged, in each case, with and without laser illumination to subtract background and dark counts from the recorded image as well as to reduce random image noise. A 500-µm-diameter aperture is placed behind each metasurface substrate to completely separate the generated holographic image (projected with a 3-mm lateral offset) from directly transmitted light leaking around the edges of the metasurface (which occupies a square area with a side length of 270 um). The physical size of the captured holographic image is determined by comparison to the image of an object of known size placed in the plane of the fluorescent white paper.

To verify that the utilized fluorescent transduction scheme is linear, images are recorded under illumination with different laser powers. The ratio of the fluorescence power integrated over the holographic image to that integrated over the image of the directly transmitted beam is found to be invariant as a function of incident laser power, over the full range of power levels used for the experiment.

XIV. Design of spin-multiplexed metasurfaces

The spin-multiplexed metasurfaces implemented in this work impart independent phase shift profiles on an incident beam depending on its handedness, namely left-handed circularly polarized (LCP) and right-handed circularly polarized (RCP). Let the LCP and RCP state be represented by Jones Vectors $|L\rangle = \begin{bmatrix} 1 \\ -i \end{bmatrix}$ and $|R\rangle = \begin{bmatrix} 1 \\ i \end{bmatrix}$, respectively, and the wave transformation characteristics of the spin-multiplexed metasurface be described by a Jones Matrix $J(x, y)$.

$J(x, y)$ then satisfies the pair of equations

$$\begin{cases} e^{i\varphi_1(x,y)}|R\rangle = J(x, y)|L\rangle \qquad (1) \\ e^{i\varphi_2(x,y)}|L\rangle = J(x, y)|R\rangle \qquad (2) \end{cases}$$

where $\varphi_1(x, y)$ and $\varphi_2(x, y)$ denote the phase shift profiles of the emerging right-handed and left-handed light, for left-handed and right-handed illumination, respectively.

The Jones Matrix $J(x, y)$ can then be written as:

$$J(x, y) = \begin{bmatrix} e^{i\varphi_1(x,y)} & e^{i\varphi_2(x,y)} \\ -ie^{i\varphi_1(x,y)} & ie^{i\varphi_2(x,y)} \end{bmatrix} \begin{bmatrix} 1 & 1 \\ -i & i \end{bmatrix}^{-1} \qquad (3)$$

Such a Jones Matrix can be realized using uniaxial (geometrically birefringent) nanostructures providing different phase shifts along their two orthogonal principle axes (here denoted as *I* and *II*)[5]. In this study, we use HfO_2 nanopillars of in-plane elliptical cross-sections to provide such birefringent functionality. To implement the Jones Matrix of Equation (3), the phase shifts experienced by the constituent linear polarization components of the circularly-polarized incident light decomposed along principle axes *I* and *II* must satisfy $\delta_I(x, y) = [\varphi_1(x, y) + \varphi_2(x, y)]/2$,

and $\delta_{II}(x,y) = -\pi + [\varphi_1(x,y) + \varphi_2(x,y)]/2$, respectively. In addition, the orientation angle $\theta(x,y)$ of the cylinder's principle axis *II* must satisfy:

$$\theta(x,y) = [\varphi_1(x,y) - \varphi_2(x,y)]/4 \qquad (4)$$

To design spin-multiplexed metasurfaces operating at λ_0 = 364 nm, the transmittance and phase shift for propagation of 364-nm-wavelength light, linearly-polarized either (1) parallel to one principle axis *I* (T_1 and Δ_1), or (2) parallel to the other principle axis *II* (T_2 and Δ_2) of an array of elliptical HfO_2 pillars (of orthogonal principle axis lengths D_1 and D_2, height *H*, and lattice spacing *P*) are computed using the finite-difference-time-domain (FDTD) simulations with periodic boundary conditions. For a given pillar height *H* and lattice spacing *P*, D_1 and D_2 are iteratively varied to identify orthogonal principle axis combinations simultaneously leading to $|\Delta_1 - \Delta_2| \approx \pi$ and $T_1 \approx T_2$, in other words, half-wave-plate-like operation. To confine the search to a computationally reasonable parameter space, the targeted values of Δ_1 are restricted to eight discrete, equally spaced values spanning the full 0 to 2π range (modulo 2π), required for arbitrary hologram implementation. The above constraints are found to be achievable with eight different nanopillar arrays, each of which are composed of elliptical nanopillars (denoted as C_1^{364} to C_8^{364}) having uniform height *H* = 500 nm, uniform spacing *P* = 330 nm, and discrete principle axis combinations, (D_1, D_2), expressed in nanometers, of (70, 190), (80, 240), (90, 260), (100, 270), (190, 70), (240, 80), (260, 90), and (270, 100), respectively (where half of the set is mathematically degenerate under an in-plane coordinate system rotation by 90°). The simulated orthogonal principle axis transmittance combinations, (T_1, T_2), are (93.4 %, 62.5 %), (93.1 %, 68.1 %), (95.6 %, 61.4 %), (89.1 %, 51.5 %), (62.5 %, 93.4 %), (68.1 %, 93.1 %), (61.4 %, 95.6 %), and (51.5 %, 89.1 %) for arrays composed exclusively of pillars C_1^{364} to C_8^{364}, respectively. The simulated orthogonal principle axis phase shift combinations, (Δ_1, Δ_2), are (0.82π, 2π), (1.35π, 0.41π), (1.58π, 0.67π), (1.87π, 0.87 π), (2π, 0.82π), (0.41π, 1.35π), (0.67π, 1.58π), and (0.87π, 1.87π) for the corresponding arrays.

To implement each spin-multiplexed metasurface operating at 364 nm (metahologram $\mathrm{H}_{364}^{\mathrm{spin}}$ and self-accelerating beam generator $\mathrm{B}_{364}^{\mathrm{spin}}$), the required phase shifts induced along the two orthogonal principle axes of an elliptical pillar of center position (x_c, y_c), $\phi_I[x_c, y_c]$ and $\phi_{II}[x_c, y_c]$, are calculated as $\phi_I[x_c, y_c] = mod(\big(\varphi_1(x_c, y_c) + \varphi_2(x_c, y_c)\big)/2\,, 2\pi)$ and $\phi_{II}[x_c, y_c] = mod(-\pi + \big(\varphi_1(x_c, y_c) + \varphi_2(x_c, y_c)\big)/2\,, 2\pi)$, respectively. ϕ_I is then compared to each element $\emptyset_{ref,i}$ of a discretized reference phase set $\emptyset_{ref} = \{\pi, 1.25\pi, 1.5\pi, 1.75\pi, 2\pi, 0.25\pi, 0.5\pi, 0.75\pi\}$, in order to identify the closest element to C_i^{364} (in other words, the index *i* such that $|\emptyset_{ref,i} - \phi_I| \leq 0.125\pi$ is satisfied). The resulting index *i* then identifies the specific nanopillar $C_i^{364} \in \{C_1^{364}, \cdots, C_8^{364}\}$ providing the optimal choice for metahologram constituent nanopillar at position (x_c, y_c). For each of such choice, the orientation angle $\theta[x_c, y_c]$ of the cylinder's principle axis *II* is set to $\theta[x_c, y_c] = mod((\varphi_1(x_c, y_c) - \varphi_2(x_c, y_c))/4\,, 2\pi)$.

Following the same design procedure, the spin-multiplexed metasurfaces operating at λ_0 = 266 nm can be implemented using a pillar library which consists of eight distinct elliptical nanopillars (denoted as C_1^{266} to C_8^{266}) having uniform height *H* = 480 nm, uniform spacing *P* = 160 nm, and eight discrete principle axis combinations, (D_1, D_2), expressed in nanometers, of (118, 58), (126, 62), (126, 70), (118, 50), (58, 118), (62, 126), (70, 126), and (118, 50) respectively (where half of the set is mathematically degenerate under an in-plane coordinate system rotation by 90°). The simulated orthogonal principle axis transmittance combinations, (T_1, T_2), are (93.4 %, 99.5 %), (94.4 %, 98.5 %), (94.8 %, 94.9 %), (97.8 %, 96.6 %), (99.5 %, 93.4 %), (98.5 %, 94.4 %), (94.9 %, 94.8 %), and (96.6 %, 97.8 %) for arrays composed exclusively of pillars C_1^{266} to C_8^{266}, respectively. The simulated orthogonal principle axis phase shift combinations, (Δ_1, Δ_2), are (1.02π, 2π), (1.21π, 0.26 π), (1.54π, 0.48π), (1.74π, 0.8 π), (2π, 1.02 π), (0.26π, 1.21π), (0.48π, 1.54π), and (0.8π, 1.74π) for the corresponding arrays. The two independent phase shift profiles associated with the spin-multiplexed metahologram, $\mathrm{H}_{266}^{\mathrm{spin}}$, are implemented by choosing, at each position of the metasurface, the optimal nanopillar $C_i^{266} \in \{C_1^{266}, \cdots, C_8^{266}\}$, according to the algorithm outlined in earlier part of this section.

XV. Characterization procedure for spin-multiplexed self-accelerating beam generator $\mathbf{B_{364}^{spin}}$

To characterize the spin-multiplexed self-accelerating beam generator B_{364}^{spin}, a normally incident, 364-nm continuous wave (CW) laser beam (beam diameter: ≈ 5 mm) is used to illuminate the device, and a linear polarizer and a quarter-wave plate (of center wavelength 355 nm) are used in addition to control the handedness of the illuminating light. The intensity distributions of the generated self-accelerating beams in selected *z*-planes beyond the metasurface are recorded using custom-built imaging system including an NA = 0.75 objective and an EMCCD camera, under LCP and RCP illumination, respectively. Specific *z*-planes (ranging from 2.5 mm to 4.5 mm, with an increment of 0.5 mm, for LCP illumination, and ranging from 4.5 mm to 10.5 mm, with an increment of 1.5 mm, for RCP illumination) are addressed using a stage which translates the metasurface relative to the camera, along the direction of the laser beam. A 500-µm-diameter aperture is placed behind the metasurface substrate to completely separate the generated self-accelerating beam from directly transmitted light leaking around the edges of the metasurface (which occupies a square area with a side length of 330 µm). The physical size of the captured intensity profile is determined by comparison to the image of the area occupied by the metasurface.

The experimental SAB generated by the device, under LCP and RCP illumination, respectively, exhibits a diffraction-free character with *xy*-plane intensity distributions similar to the targeted intensity distributions (Figs. S14 and S15), numerically computed using the angular spectrum representation method[4], assuming an ideal metasurface realization having both the designed phase shift profile φ^B and an unity transmittance T.

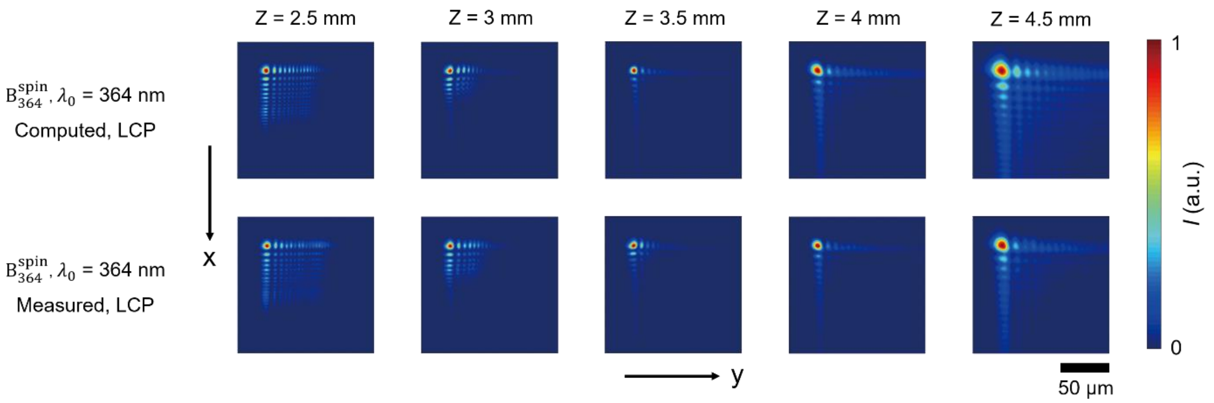

Fig. S14. Computed and measured *xy*-plane intensity distributions (normalized) at different *z* planes for device B_{364}^{spin} at its designated wavelength of operation under LCP illumination. Each distribution is displayed over an equal square area with a side length of 140 μm, but shifted along the -*xy* direction as a function of increasing *z*, such that the center of the main lobe maintains an invariant position within each image.

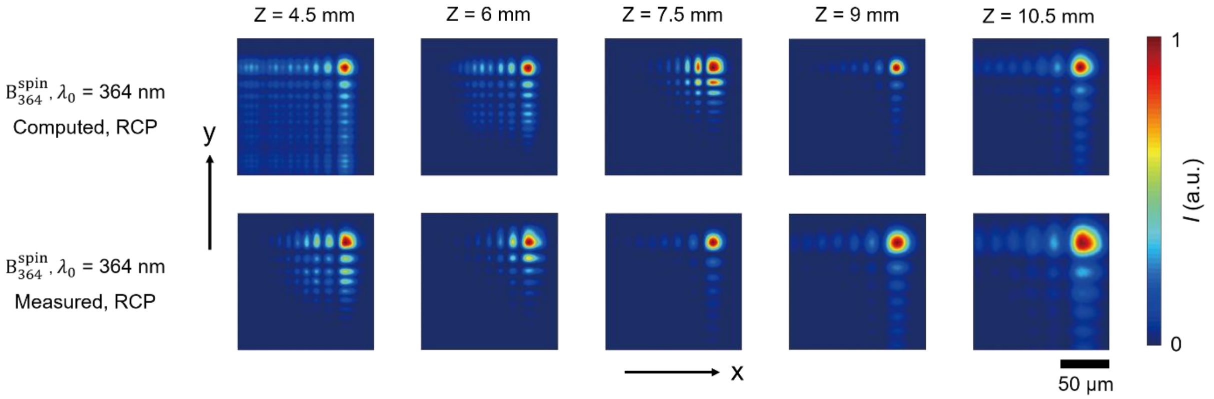

Fig. S15. Computed and measured *xy*-plane intensity distributions (normalized) at different *z* planes for device B_{364}^{spin} at its designated wavelength of operation under RCP illumination. Each distribution is displayed over an equal square area with a side length of 140 μm, but shifted along the *xy* direction as a function of increasing *z*, such that the center of the main lobe maintains an invariant position within each image.

XVI. Discussion of reduced device efficiency under LCP illumination for $\mathbf{B_{364}^{spin}}$

The spin-multiplexed SAB generator, B_{364}^{spin}, occupies a square area with a side length of l = 330 µm. Two distinct phase shift profiles, $\varphi^{LCP}(x,y,\lambda_0) = mod\left(-\frac{8\pi}{3\lambda_0}\sqrt{16}\left(x^{\frac{3}{2}} + y^{\frac{3}{2}}\right), 2\pi\right)$ and $\varphi^{RCP}(x,y,\lambda_0) = mod\left(-\frac{8\pi}{3\lambda_0}\sqrt{2.25}\left((l-x)^{\frac{3}{2}} + (l-y)^{\frac{3}{2}}\right), 2\pi\right)$, are targeted for device operation, in order to yield SABs exiting the metasurface from opposite corners and following different trajectories, $y = x = -d_1 = -16z^2$, and $(y-l) = (x-l) = d_2 = 2.25z^2$, under LCP and RCP illumination, respectively.

The targeted phase shift profiles for LCP and RCP illuminations, $\varphi^{LCP}(x,y)$ and $\varphi^{RCP}(x,y)$, are plotted in Fig. S16. For the purpose of clarity, the phase shift profiles are only displayed over a 50 µm by 50 µm square area with one corner at (0, 0) and (330 µm, 330 µm) for φ^{LCP} and φ^{RCP}, respectively. It can be easily seen that φ^{LCP} exhibits a higher spatial gradient than φ^{RCP} and varies rapidly over the space.

The implemented metasurface consists of nanopillars of elliptical in-plane cross-sections, that are arrayed in a square lattice with lattice spacing of 330 nm. The size and orientation of each nanopillar is chosen based on the targeted values of φ^{LCP} and φ^{RCP} in the center position of the lattice, as well as the associated design library. Consequently, the spatially-continuously-varying phase shift profiles, $\varphi^{LCP}(x,y)$ and $\varphi^{RCP}(x,y)$, are now realized (approximated) by discrete phase values over the metasurface plane. Due to its higher spatial gradient (faster spatial variation), φ^{LCP} is less ideally implemented compared to φ^{RCP}, and this leads to the reduced metasurface efficiency under LCP illumination.

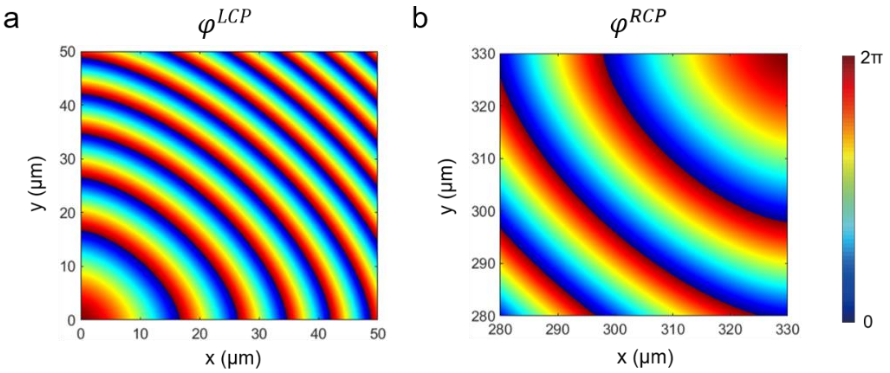

Fig. S16. Targeted phase shift profiles, $\varphi^{LCP}(x, y)$ and $\varphi^{RCP}(x, y)$, for the spin-multiplexed SAB generator. For the purpose of clarity, the phase shift profiles are only displayed over a 50 µm by 50 µm square area with one corner at (0, 0) and (330 µm, 330 µm) for φ^{LCP} and φ^{RCP}, respectively.

XVII. Implementation of spin-multiplexed metahologram H_{364}^{spin}

A spin-multiplexed metahologram, H_{364}^{spin}, occupying a square area of side length 330 μm, is implemented for operation at λ_0 = 364 nm. Different phase profiles (Fig. S17), $\varphi_{364,LCP}^{H,spin}(x, y, \lambda_0)$ and $\varphi_{364,RCP}^{H,spin}(x, y, \lambda_0)$, all based on the Gerchberg-Saxton algorithm, are calculated for projecting a holographic "NIST" image (for LCP illuminating light) and "NJU" image (for RCP illuminating light), all located in the z = 40 mm plane, with an offset of $y = -3$ mm (Fig. S18).

A normally incident, 364-nm continuous wave (CW) laser beam (beam diameter: ≈ 5 mm) is used to illuminate the metahologram, and a linear polarizer and a quarter-wave plate (with a center wavelength of 355 nm) are used in addition to control the handedness of the illuminating light. An EMCCD camera is placed in the hologram formation plane located 40 mm beyond the metahologram to directly record the projected holographic images under LCP and RCP illumination, respectively. A 500-μm-diameter aperture is placed behind the metahologram substrate to completely separate the generated holographic image (projected with a 3-mm lateral offset) from directly transmitted light leaking around the edges of the metasurface (which occupies a square area with a side length of 330 μm).

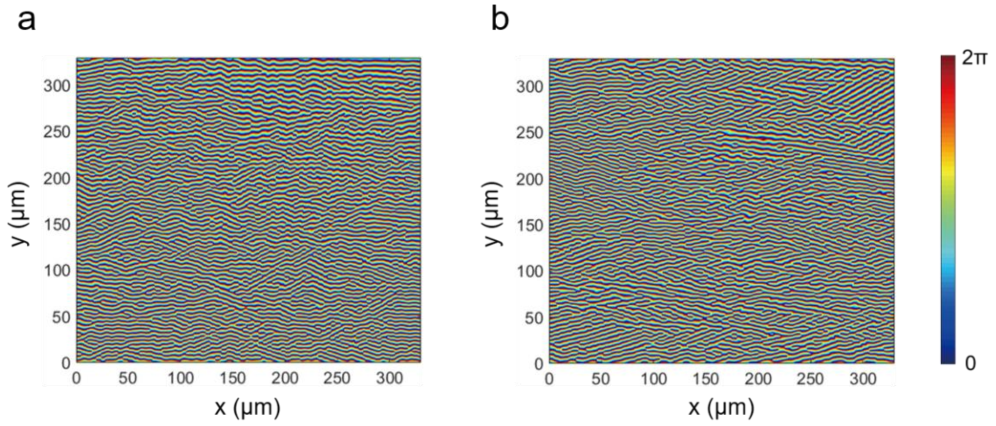

Fig. S17. Metasurface phase shift profiles, $\varphi^{\mathrm{H,spin}}_{364,\mathrm{LCP}}(\mathrm{x},\mathrm{y},\lambda_0)$ and $\varphi^{\mathrm{H,spin}}_{364,\mathrm{RCP}}(\mathrm{x},\mathrm{y},\lambda_0)$, designed to project a "NIST" (a) and "NJU" (b) holographic image in the *z* = 40 mm plane, under normal-incidence, plane-wave illumination at λ_0 = 364 nm (implemented by the spin-multiplexed metahologram $\mathrm{H}^{\mathrm{spin}}_{364}$).

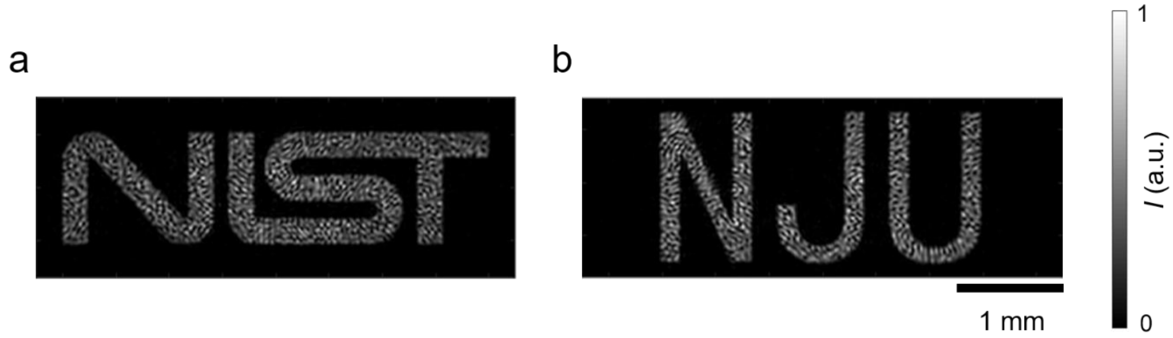

Fig. S18. Targeted holographic images in the *z* = 40 mm plane, numerically computed assuming an ideal metahologram realization having an unity transmittance, and either (a) phase shift profile $\varphi^{\mathrm{H,spin}}_{364,\mathrm{LCP}}(x,y,\lambda_0)$ shown in Fig. S17a or (b) phase shift profile $\varphi^{\mathrm{H,spin}}_{364,\mathrm{RCP}}(x,y,\lambda_0)$ shown in Fig. S17b, under normal-incidence, plane-wave illumination at λ_0 = 364 nm.

XVIII. Implementation of spin-multiplexed metahologram $\mathbf{H}_{\mathbf{266}}^{\mathbf{spin}}$

Two different phase shift profiles over 320-μm-square areas (Fig. S19), $\varphi_{266,\mathrm{LCP}}^{\mathrm{H,spin}}(x, y, \lambda_0)$ and $\varphi_{266,\mathrm{RCP}}^{\mathrm{H,spin}}(x, y, \lambda_0)$, are calculated using the Gerchberg-Saxton algorithm to achieve projection of distinct holographic images under normal-incidence, plane-wave illumination at λ_0 = 266 nm, namely, "deep" and "UV" images, respectively, in the *z* = 40 mm plane, with an offset $y = -3$ mm (Fig. S20).

A normally incident, 266-nm pulsed laser (pulse duration: ≈ 5 ns, repetition rate: 10 Hz, beam diameter: ≈ 6 mm) is used to illuminate the spin-multiplexed metahologram $\mathrm{H}_{266}^{\mathrm{spin}}$. In addition, a linear polarizer and a quarter-wave plate (with a center wavelength of 266 nm) are used to control the handedness of the illuminating light. A piece of fluorescent white paper is placed in the hologram formation plane located 40 mm beyond the metahologram and imaged with a custom-built imaging system including a lens and a CCD camera. Holographic images are recorded for device $\mathrm{H}_{266}^{\mathrm{spin}}$ under LCP and RCP illumination, respectively. 500 images are recorded and averaged, in each case, with and without laser illumination to subtract background and dark counts from the recorded image as well as to reduce random image noise. A 500-μm-diameter aperture is placed behind each metasurface substrate to completely separate the generated holographic image (projected with a 3-mm lateral offset) from directly transmitted light leaking around the edges of the metasurface (which occupies a square area of side length 320 μm). The physical size of the captured holographic image is determined by comparison to the image of an object of known size placed in the plane of the fluorescent white paper.

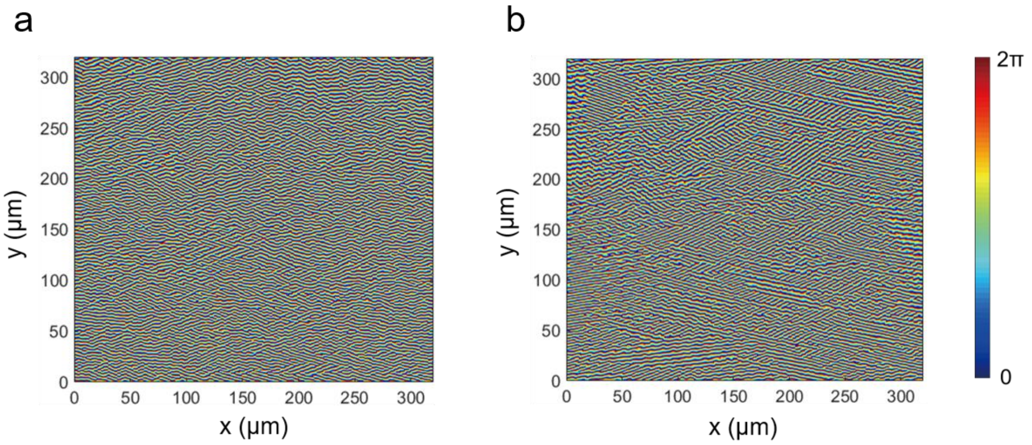

Fig. S19. Metasurface phase shift profiles, $\varphi_{266,\mathrm{LCP}}^{\mathrm{H,spin}}(x, y, \lambda_0)$ and $\varphi_{266,\mathrm{RCP}}^{\mathrm{H,spin}}(x, y, \lambda_0)$, designed to project a "deep" (a) and "UV" (b) holographic image in the *z* = 40 mm plane, under normal-incidence, plane-wave illumination at λ_0 = 266 nm (implemented by spin-multiplexed metahologram $\mathrm{H}_{266}^{\mathrm{spin}}$).

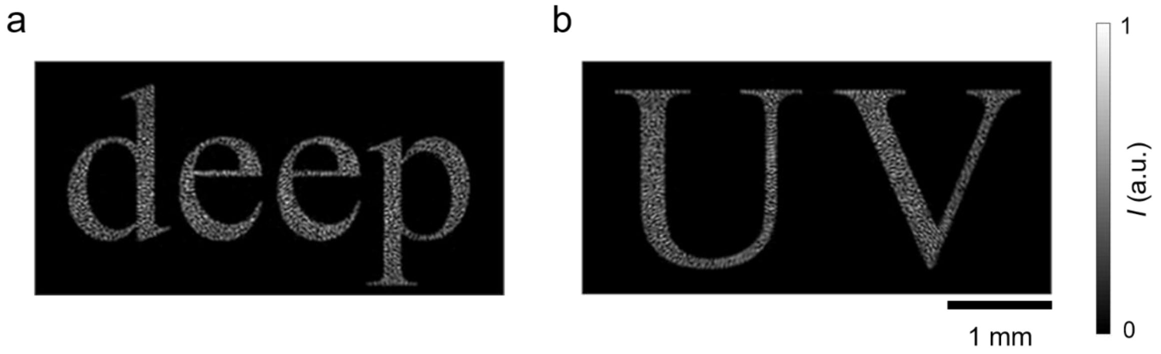

Fig. S20. Targeted holographic images in the *z* = 40 mm plane, numerically computed assuming an ideal metahologram realization having an unity transmittance, and either (a) phase shift profile $\varphi_{266,\mathrm{LCP}}^{\mathrm{H,spin}}(x, y, \lambda_0)$ shown in Fig. S19a or (b) phase shift profile $\varphi_{266,\mathrm{RCP}}^{\mathrm{H,spin}}(x, y, \lambda_0)$ shown in Fig. S19b, under normal-incidence, plane-wave illumination at λ_0 = 266 nm.

References:

1. Hilfiker, J. N., Singh, N., Tiwald, T., Convey, D., Smith, S. M., Baker, J. H., Tompkins, H. G. Survey of methods to characterize thin absorbing films with Spectroscopic Ellipsometry. *Thin Solid Films* **516**, 7979 (2008).

2. Zhang, C., Hong, N., Ji, C., Zhu, W., Chen, X., Agrawal, A., Zhang, Z., Tiwald, T. E., Schoeche, S., Hilfiker, J. N., Guo, L. J., Lezec, H. J. Robust extraction of hyperbolic metamaterial permittivity using total internal reflection ellipsometry. *ACS Photonics* **5**, 2234 (2018).

3. Novotny, L., Hecht, B. Principles of Nano-Optics Ch. 3 (Cambridge Univ. Press, Cambridge, 2006).

4. Gerchberg, R. W., Saxton, W. O. A practical algorithm for the determination of the phase from image and diffraction plane pictures. *Optik* **35**, 237 (1972).

5. Balthasar Mueller, J. P., Rubin, N. A., Devlin, R. C., Groever, B., Capasso, F. Metasurface polarization optics: independent phase control of arbitrary orthogonal states of polarization. *Physical Review Letters* **118**, 113901 (2017).
